# Genome-Wide Identification and Expression Analysis of Aquaporins in Tomato

**DOI:** 10.1371/journal.pone.0079052

**Published:** 2013-11-19

**Authors:** Stefan Reuscher, Masahito Akiyama, Chiharu Mori, Koh Aoki, Daisuke Shibata, Katsuhiro Shiratake

**Affiliations:** 1 Graduate School of Bioagricultural Sciences, Nagoya University, Chikusa, Nagoya, Japan; 2 Graduate School of Life and Environmental Sciences, Osaka Prefecture University, Gakuen-cho, Sakai, Japan; 3 Kazusa DNA Research Institute, Kazusa-kamatari, Kisarazu, Japan; Rosalind Franklin University, United States of America

## Abstract

The family of aquaporins, also called water channels or major intrinsic proteins, is characterized by six transmembrane domains that together facilitate the transport of water and a variety of low molecular weight solutes. They are found in all domains of life, but show their highest diversity in plants. Numerous studies identified aquaporins as important targets for improving plant performance under drought stress. The phylogeny of aquaporins is well established based on model species like *Arabidopsis thaliana*, which can be used as a template to investigate aquaporins in other species. In this study we comprehensively identified aquaporin encoding genes in tomato (*Solanum lycopersicum*), which is an important vegetable crop and also serves as a model for fleshy fruit development. We found 47 aquaporin genes in the tomato genome and analyzed their structural features. Based on a phylogenetic analysis of the deduced amino acid sequences the aquaporin genes were assigned to five subfamilies (PIPs, TIPs, NIPs, SIPs and XIPs) and their substrate specificity was assessed on the basis of key amino acid residues. As ESTs were available for 32 genes, expression of these genes was analyzed in 13 different tissues and developmental stages of tomato. We detected tissue-specific and development-specific expression of tomato aquaporin genes, which is a first step towards revealing the contribution of aquaporins to water and solute transport in leaves and during fruit development.

## Introduction

Water is an essential substance for all life on earth. Adequate supply with water is critical for plants to thrive. In agriculture and horticulture water supply is critically to achieve high yields. Approximately 70% of all fresh water use in the world can be attributed to agriculture, with developing countries using up to 95% of their water resources for the irrigation of crops (www.faostat.org). One fifth of the word population is already living under conditions of water scarcity and with increasing population that number will increase in the future [1]. Given the importance of irrigation for agriculture, uptake and transport, and ultimately efficiency of water use, are important subjects of study.

The primary uptake organ of plants for water is the root, and in order to bypass the Casparian strip and reach the xylem water has to cross the plasma membrane (PM) and enter the symplast. Since biomembranes are essentially a lipid bilayer, they present an obstacle for water uptake. Also within the plant efficient cell-to-cell transport of water is needed for growth and development. To achieve this specialized channel proteins are present in the membranes of not only plants but all living organisms. Aquaporins (AQPs) are water channel proteins that allow rapid and selective transport of water across membranes. They were first discovered in human erythrocytes [2] and plant nodules associated with N fixation [3]. Since then it became clear that AQPs belong to a large family of channel proteins called major intrinsic proteins (MIPs) [4]. The MIP family is comprised of AQPs in the strict sense, which are water transporters, and also aquaglyceroproteins which facilitate the transport of a variety of solutes, like B, NH_4_^+^, glycerol or urea. Water movement through the plant is controlled by AQPs in different physiological contexts [5]. In addition to a role in water uptake into the roots, AQPs also play a role in water homeostasis in the leaf [6], [7]. Finally, AQPs are implicated in controlling water movement during tissue expansion [8], [9].

The classification based on sequence comparison of plant AQPs is well established. There are currently five major subfamilies recognized in plants based on sequence similarities. The plasma membrane intrinsic proteins (PIPs), the tonoplast intrinsic proteins (TIPs), the NOD26-like intrinsic proteins (NIPs), the small basic intrinsic proteins (SIPs) [10] and the plant-specific subfamily of X-intrinsic protein (XIPs) [11], [12]. Although the subfamilies were originally named after the subcellular localization of its members, it was shown that this classification does not always represent the actual localization [13]. In humans 13 different AQPs have been identified [14]. In contrast to this, the AQP family comprises more members in the plant kingdom. There were 35 AQPs found in *Physcomitrella patens* [12] and *Arabidopsis thaliana* [15], [16], 66 in *Glycine max* [17], 71 in *Gossypium hirsutum* [18], 54 in *Populus trichocarpa* [19], [20], 31 in *Zea mays* [21] and *33* in *Oryza sativa* [22].

Tomato is important not only as a vegetable crop from a commercial point of view but also as a model to study fruit physiology in basic research. A lot of information about tomato, including EST and full-length cDNA information can be obtained from databases such as the Sol Genomics Network (http://www.solgenomics.net/) and TOMATOMICS (http://www.bioinf.mind.meiji.ac.jp/tomatomics/) [23]. Also transcriptome data (at TOMATOMICS) and metabolome data of Solanaceae species (KaPPA-View4 SOL at http://www.kpv.kazusa.or.jp/kpv4-sol/) are available. A dwarf variety of tomato, called ‘Micro-Tom’ is used as a model for tomato genetics and physiology because of its small size and shorter generation time compared to commercial cultivars [24]. Ethylmethanesulfonate and gamma ray irradiation-induced mutant lines of Micro-Tom have been generated and are available from TOMATOMA (http://www.tomatoma.nbrp.jp/index.jsp) [25].

A high-quality genome sequence of the commercial tomato cultivar ‘Heinz 1706’ became available recently [26]. This enabled us to comprehensively study the family of tomato AQPs. We were able to detect a total of 47 genes putatively encoding AQPs. Taking into account the nomenclature proposed by Sade *et al.* 2009 [27] for tomato AQPs and nomenclature used in other plant species we assigned all 47 genes to established subfamilies. To provide a comprehensive overview of all members we analyzed exon-intron structure as well as conserved residues putatively determining substrate specificity. Also subcellular localizations and transmembrane domains were predicted. To select single AQPs for future research, expression analysis was performed in vegetative tissues and during fruit development.

## Materials and Methods

### Identification of *Solanum lycopersicum* AQPs

To comprehensively identify *Solanum lycopersicum* AQPs the tomato genome was analyzed using the BLAST tools available from the Sol Genomics Network (http://www.solgenomics.net) [28]. For each of the five tomato AQP subfamilies, the CDS (coding DNA sequence) of an already identified tomato AQP was used as a query to identify additional members from the complete set of predicted CDSs (ITAG release 2.3 SL2.40) [26]. The identified CDSs were then used to find cDNAs and EST clones from the EST databases found at http://www.pgb.kazusa.or.jp/mibase [29] or http://www.solgenomics.net. After consolidation of the data, the most similar EST clone for each putative AQP locus was obtained and sequenced to verify the current gene model. All EST sequences are available from the DNA Data Bank of Japan (http://www.ddbj.nig.ac.jp/) under the accession numbers AB845604 to AB845638.

### Multiple sequence alignments and phylogenetic analysis

Final classification of AQP genes into subfamilies and subgroups was done according to phylogenetic analysis. Multiple sequence alignments using the predicted AA (amino acid) sequences were made using the CLUSTAL alignment function in the CLC Main Workbench software (CLC Bio, Aarhus, Denmark). Phylogenetic trees were built using the Neighbor-joining algorithm in the same software and visualized using Treeview [30] and Dendroscope [31].

### *In silico* prediction of subcellular localization and transmembrane helical domains

Prediction of subcellular localization of putative AQPs was performed using the WoLFPSORT algorithm (http://wolfpsort.seq.cbrc.jp) [32]. Prediction of transmembrane helical domains was performed using TMHMM Server v.2.0 (http://www.cbs.dtu.dk/services/TMHMM/) [33].

### Plant material and growth conditions

*Solanum lycopersicum* plants for gene expression analysis were of the dwarf cultivar ‘Micro-Tom’. Plants were grown on soil in a growth chamber (Biotron LPH-350S, NK Systems, Osaka, Japan) with a light regime of 8 h of light/16 h darkness at 25°C and 60% relative humidity. Plants were watered twice a week with tap water. Fertilizer (Otsuka Chemicals, Osaka, Japan) was applied once per week.

### RNA isolation and cDNA synthesis

Plant tissues from young leaves, mature leaves, roots, shoots, flowers and from developing fruits 3, 7, 14, 21 and 28 days after pollination (DAP) and during the Breaker, Orange and Red stages of fruit development were harvest into liquid nitrogen. Vegetative tissues were harvested from *ca.* six week old plants. Samples of young leaves included developing, not fully expanded leaves, samples of mature leaves included fully expanded, non-senescent leaves. RNA from developing fruits 14 and 21 DAP was isolated using the RNA Suisui-R kit (Rizo, Tsukuba, Japan). RNA from all other tissues was isolated using TRIzol reagent (Life Technologies, Carlsbad, USA) following the manufacturer's protocol. Quality of the RNA was assessed using a spectrophotometer. RNA was stored at −80°C. cDNA was prepared using the PrimeScript RT reagent Kit with gDNA Eraser (Clontech, Mountain View, USA) according to the manufacturer's protocol. For each 20 µl reaction 500 ng of total RNA was used.

### RT-PCR expression analysis

Semi-quantitative RT-PCR was performed using 0.1 µl cDNA preparation as a template and EmeraldAmp PCR Mastermix (Clontech, Mountain View, USA) for all other components needed for PCR. For each primer pair the PCR program was empirically adjusted (Table S1). All primers were tested for specificity by trying to obtain a PCR product using plasmid DNA containing ESTs from other subfamily members as a template (data not shown). As an internal control the constitutively expressed gene *SlUBQ* (Ubiquitin, Solyc01g056940.1) was used. PCR products were analyzed using 1% (w/v) Agarose gels stained for nucleic acids with Ethidium Bromide.

## Results and Discussion

### Genome-wide identification of *Sl*AQPs

By using identified tomato AQP sequences as queries we could detect 47 loci in the tomato genome putatively encoding AQPs (Table 1). This number is consistent with the number of AQPs found in the genome of other plant. For 36 of these loci at least one EST was found. It is possible that the 11 loci with no EST evidence are pseudogenes or are expressed exclusively in response to a specific stimulus or in a very specific part of the plant and thus are not represented in the available EST collections. In some cases the DNA sequence of the EST revealed slightly different splicing compared to the predicted gene model for the respective locus. In these cases the experimentally determined sequence was used for further analysis. In two cases (*SlPIP2;12* and *SlXIP1;2*) the sequenced ESTs had a 1 bp insertion compared to the reference genome, leading to a frameshift and a premature stop codon. We assumed these insertions were artifacts from EST cloning and used corrected, full-length ORFs for our further analysis.

**Table 1:** Comprehensive nomenclature and feature list of 47 aquaporins identified in the tomato genome.

	Gene Name	Locus	Best Hit EST	DDBJ No.	AA^1^	TMD^2^	Comments
PIP	*SlPIP1;1* §	Solyc08g008050.2	SGN-E310188	AB845604	288	6	
	*SlPIP1;2* §	Solyc01g094690.2	LEFL1005BF02	AB845605	286	6	
	*SlPIP1;3* §	Solyc12g056220.1	LEFL1045BE12	AB845606	289	6	
	*SlPIP1;5* §	Solyc08g081190.2	LEFL1015BC05	AB845607	287	6	
	*SlPIP1;7* §	Solyc03g096290.2	FC17CC02	AB845608	287	6	
	*SlPIP2;1* §	Solyc09g007770.2	FC04BE01	AB845609	280	6	
	*SlPIP2;4* §	Solyc06g011350.2	LEFL1052AA02	AB845610	281	6	
	*SlPIP2;5* §	Solyc10g084120.1	SGN-E542248	AB845611	282	6	
	*SlPIP2;6* §	Solyc11g069430.1	FC11CE01	AB845612	288	6	
	*SlPIP2;8* §	Solyc01g111660.2	LEFL1010CC03	AB845613	284	6	
	*SlPIP2;9* §	Solyc10g055630.1	LEFL1088BC11	AB845614	284	6	
	*SlPIP2;10*	Solyc09g007760.2	Not Found	-	pred. 307	6	
	*SlPIP2;11*	Solyc02g083510.2	Not available#	-	pred. 260	6	short N- and C- terminus
	*SlPIP2;12*	Solyc05g055990.2	LEFL1068CF11	AB845615	274	5	EST frameshift*
TIP	*SlTIP1;1* §	Solyc06g074820.2	FC01AB01	AB845616	251	7	
	*SlTIP1;2* §	Solyc06g075650.2	SGN-E544724	AB845617	254	6	
	*SlTIP1;3*	Solyc10g083880.1	Not Found	-	pred. 249	7	
	*SlTIP2;1* §	Solyc12g044330.1	LEFL1025BD07	AB845618	249	7	
	*SlTIP2;2* §	Solyc03g120470.2	LEFL1013DH10	AB845619	250	7	characterized in [27]
	*SlTIP2;3* §	Solyc06g060760.2	LEFL1068BB11	AB845620	251	6	
	*SlTIP2;5*	Solyc06g066560.1	SGN-E545679	AB845621	274	7	EST not full length (Δ1–22)
	*SlTIP3;1* §	Solyc06g072130.2	FC17BG08	AB845622	260	6	EST not full length (Δ1–76)
	*SlTIP3;2* §	Solyc03g019820.2	FC17AH05	AB845623	261	6	
	*SlTIP4;1* §	Solyc08g066840.2	FC02AF05	AB845624	248	6	
	*SlTIP5;1*	Solyc03g093230.2	Not Found	-	pred. 252	6	
NIP	*SlNIP1;1* §	Solyc03g005980.2	SGN-E351875	AB845625	278	6	EST not full length (Δ1–173)
	*SlNIP1;2*	Solyc02g071920.2	LEFL1060CF11	AB845626	291	6	
	*SlNIP2;1* §	Solyc03g013340.2	LEFL1026AC05	AB845627	284	6	
	*SlNIP2;2*	Solyc02g071910.1	Not Found	-	pred. 232	4	17 AA from TMD2 deleted
	*SlNIP3;1*	Solyc06g073590.2	LEFL3101K20	AB845628	346	6	
	*SlNIP3;2*	Solyc12g057050.1	Not Found	-	pred. 261	5	
	*SlNIP4;1* §	Solyc02g091420.2	SGN-E361487	AB845629	268	6	
	*SlNIP4;2*	Solyc05g008080.1	Not Found	-	pred. 273	6	
	*SlNIP4;3*	Solyc02g063310.2	Not Found	-	pred. 138	5	short N- and C-terminus
	*SlNIP5;1* §	Solyc08g013730.2	LEFL2003BD12	AB845630	296	6	
	*SlNIP6;1* §	Solyc03g117050.2	LEFL1034DB12	AB845631	307	6	
	*SlNIP7;1* §	Solyc01g079890.2	SGN-E321420	AB845632	287	3	
SIP	*SlSIP1;1* §	Solyc12g019690.1	LEFL2041K14	AB845633	243	5	
	*SlSIP1;2* §	Solyc10g078490.1	LEFL1029CD02	AB845634	244	5	
	*SlSIP1;3*	Solyc10g078500.1	Not Found	-	pred. 105	2	short C-terminus
	*SlSIP2;1* §	Solyc01g056720.2	LEFL2043B16	AB845635	241	6	
XIP	*SlXIP1;1* §	Solyc10g054840.1	LEFL1059DD06	AB845636	328	6	*SlXIP1;1α* from [11]
	*SlXIP1;2*	Solyc10g054820.1	LEFL1004BA01	AB845637	248	6	EST frameshift*
	*SlXIP1;3*	Solyc10g054810.1	LEFL1078DB07	AB845638	303	6	
	*SlXIP1;4*	Solyc10g054800.1	Not Found	-	pred. 328	7	
	*SlXIP1;5*	Solyc10g054790.1	Not Found	-	pred. 329	7	
	*SlXIP1;6*	Solyc01g111010.2	Not Found	-	pred. 521	6	extended N-terminus

1 The amino acid sequence length was either confirmed by cDNA sequencing or predicted using SL2.40 gene models.

2 The number of transmembrane domains was predicted by TMHMM Server v2.0.

* The sequenced cDNA contained a 1 bp insertion (assumed to be a cloning artifact) leading to a frameshift. Further analyses were performed using the corrected gene model.

# EST is present in the databases but was not available for ordering.

§ First named by Sade *et al.*, 2007 [27].

While mostly following the nomenclature of Sade et al. [27] some AQPs identified solely on the basis of EST evidence by Sade et al. could not be integrated into our nomenclature which is based on the tomato reference genome. To avoid confusion we decided to not reuse gene names proposed by Sade et al. for these AQPs, which explains why the numeration of AQPs is not always consecutive in our nomenclature. Specifically, this affected *SlPIP1;4* and *SlPIP1;6* (ESTs BP888840 and BP876517), where a BLAST search revealed that both of these ESTs most likely belong to *SlPIP1;5* together with LEFL1015BC05 which we used to define *SlPIP1;5*. For *SlPIP2;3* (TC174068) the best BLAST hit was Soly04g0515002.1, a non AQP-type transporter. A BLAST search using *SlPIP2;7* (CO751218) did not produce a significant alignment with any annotated cDNA, while for *SlTIP2;4* (TC188024) no sequence data could be obtained from any database.

Prediction of TMDs (transmembrane domains) showed that most identified putative AQPs contained six TMDs (Table 1). Manual inspection of hydrophobicity plots (data not shown) and AA sequence alignments (Figs. S1 to S5) revealed that most likely all full-length AQPs (excluding the truncated AQPs *Sl*NIP2;2, *Sl*NIP4;3 and *Sl*SIP1,3) possess six TMDs. It is conceivable that the TMHMM algorithm did not correctly identify all TMDs. An additional analysis using the SOSUI program (data not shown) established all *Sl*AQPs as transmembrane proteins except *Sl*TIP3;2 and *Sl*SIP2;1 (http://bp.nuap.nagoya-u.ac.jp/sosui/) [34]. Similar to TMHMM, also SOSUI predicted six TMDs for most, but not all AQPs. Since the *in silico* predictions presented here are in a few cases contradicting, they should be validated by experimental means. Given the high degree of sequence conservation between AQPs it is however very likely that tomato AQPs feature six TMDs, comparable to AQPs found in other organisms.

Analysis of the predicted subcellular localization showed diverse results (data not shown), not always in agreement with experimentally determined localizations (reviewed in [35]). In summary, *Sl*PIPs were predicted to localize to the PM, which is in agreement with current literature. TIP-type AQPs were experimentally determined to localize to the tonoplast but diverse results were obtained when trying to predict *Sl*TIP localizations, including clearly mispredicted cytosolic localizations. NIP-type AQPs were determined to localize to the PM, the ER membrane or the peribacteroid membrane of root nodules in other organisms. Our *in silico* predictions included the PM, the tonoplast and chloroplast membranes. *Sl*SIPs were predicted to localize to the tonoplast, but experimental evidence showed that the *Arabdopsis Sl*SIPs are localized to intracellular membranes, most likely representing the ER [36]. Of the XIPs, *Sl*XIP1;1 was localized to the PM [11]. The other *Sl*XIPs were predicted to also localize to the PM or were mispredicted to be cytosolic or nuclear proteins.

**Figure 1:**
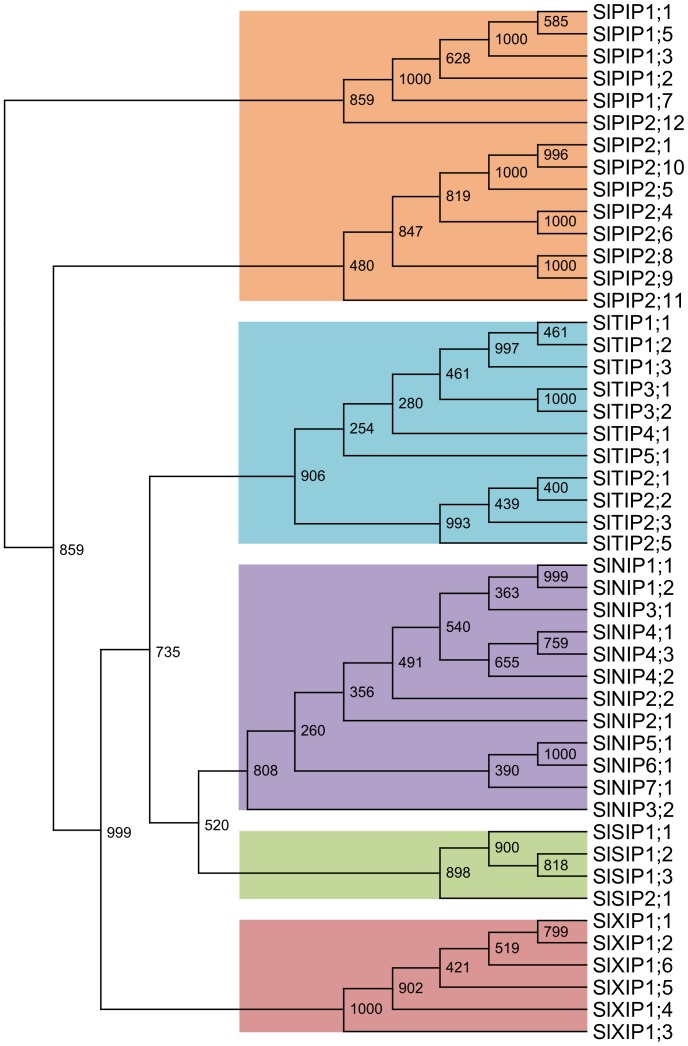
Phylogenetic analysis of 47 aquaporins identified in tomato. Shown is phylogenetic tree generated by the neighbor-joining method derived from a CLUSTAL alignment of amino acid sequences from all 47 aquaporins identified in tomato. Numbers at internal nodes show the results of bootstrapping analysis (*n* = 1000).

**Figure 2:**
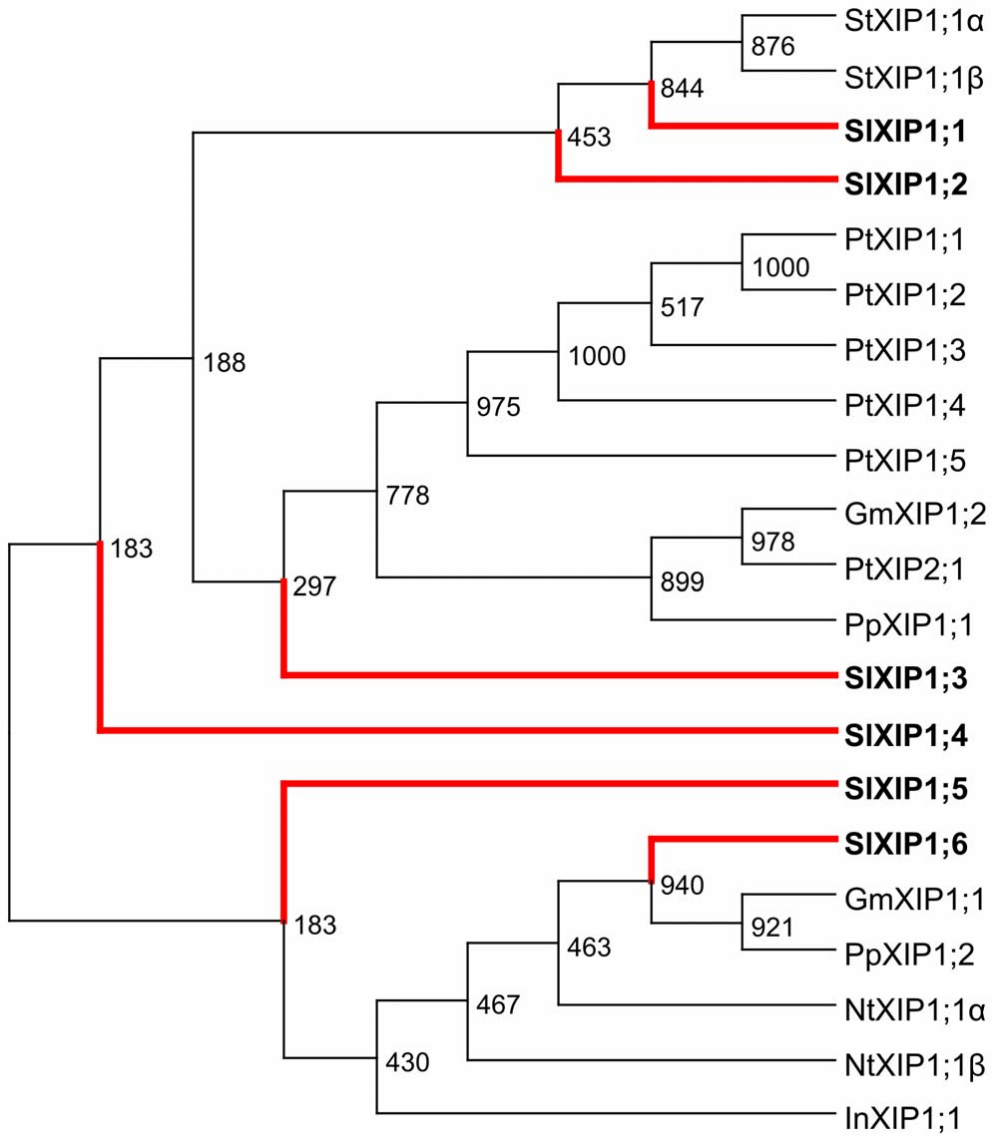
Phylogenetic analysis of XIP-family members. Shown is a phylogenetic tree generated by the neighbor-joining method derived from a CLUSTAL alignment of amino acid sequences from tomato (this study, red lines, bold type), tobacco NtXIP1;1α (HM475295), NtXIP1;1 β (HM475294), potato StXIP1;1α (HM475297), StXIP1;1β (HM475298) and morning glory InXIP1;1 (HM475296) from [11], *Physcomitrella patens* PtXIP1;1 (71087) and PtXIP1;2 (71489) from [12], soybean GmXIP1;1 (Glyma11g10360) and GmXIP1;2 (Glyma12g02640) from [17] and poplar PtXIP1;1 (829126), PtXIP1;2 (557139), PtXIP1;3 (759781), PtXIP1;4 (767334), PtXIP1;5 (821124) and PtXIP2;1 (557138) from [19]. Numbers at internal nodes show the results of bootstrapping analysis (*n* = 1000).

Through phylogenetic analysis the 47 tomato AQPs were classified into 14 PIPs, 11 TIPs, 12 NIPs, 4 SIPs and 6 XIPs (Fig. 1 and Fig. S6). Through alignments of AA sequences from members of each subfamily alone several sub-groups were found in agreement with current literature (Figs. S1 to S5). The *Sl*PIPs could be divided entirely in a *Sl*PIP1 (five members) and a *Sl*PIP2 (nine members) subgroup according to differences in their AA sequence, especially in the N- and C-terminal regions that seemed to have different water transport activities in oocyte experiments [35], [37]. Similarly, the *Sl*TIPs clustered into subgroups *Sl*TIP1 (three members), *Sl*TIP2 (three members), *Sl*TIP3 (two members) and two further *Sl*TIPs. The *Sl*NIPs were classified into *Sl*NIP1, *Sl*NIP2, *Sl*NIP3 (two members each), *Sl*NIP4 (three members) and three additional loci. In the *Sl*SIP subfamily the *Sl*SIP1 subgroup (three members) was found to form a clade distinct from *Sl*SIP2;1. The XIP-type AQPs represent a novel clade of AQPs, first described in the moss *Physcomitrella patens* [12]. Additionally, XIPs have been described in poplar [19], [20] and in selected *Solanaceae* species, including tomato [11]. A separate phylogenetic analysis using the tomato XIPs described in this study as well as the XIPs described in the literature was performed (Fig. 2). *Sl*XIP1;1 and 1;2 were found to be most similar to the two splice variant of potato *St*XIP1 described in [11]. *Sl*XIP1;5 and 1;6 were found to cluster together with XIPs from other *Solanaceae* species (tobacco and morning glory) used in this analysis, although some of the nodes were not well supported by bootstrapping analysis. It should be noted that all *Sl*XIPs, except *Sl*XIP1;6 are likely the results of recurring gene duplications, since the loci *Sl*XIP1;1 to 1;5 are found next to each other on chromosome 10. Also obvious gene duplications occurred in other subfamilies leading to the gene-pairs *SlPIP2;1*/*SlPIP2;10*, *SlNIP1;2*/*SlNIP2;2* and *SlSIP1;2*/*SlSIP1;3*.

### Analysis of exon-intron structure

**Figure 3:**
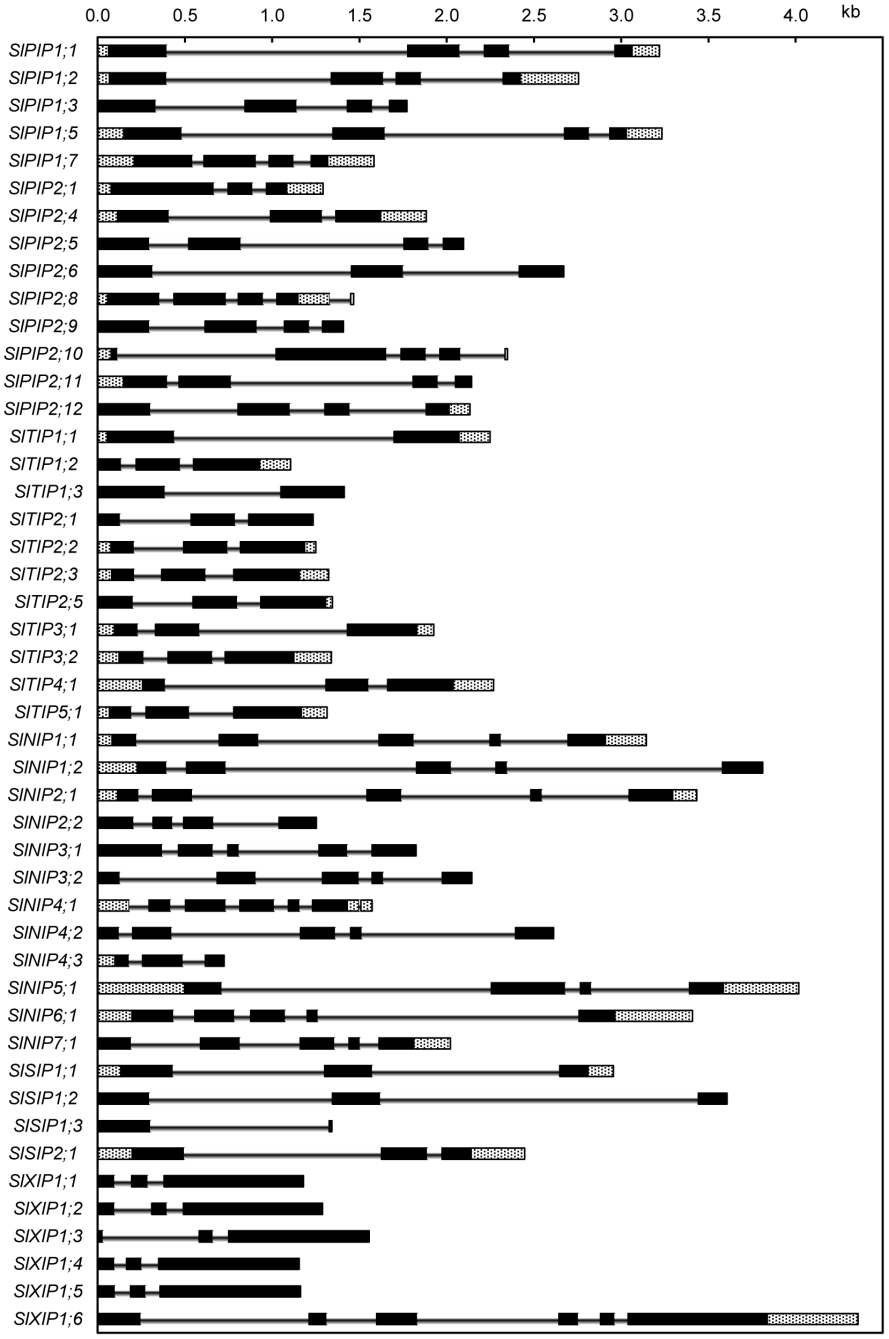
Exon-Intron structure of 47 tomato aquaporins genes. Shown is a graphic representation of the gene models of all 47 aquaporins identified in this study. UTRs are shown as hatched boxes, exons are shown as black boxes and introns are shown as black lines. Gene models are based on sequenced cDNAs. In the case of lacking cDNA evidence *in silico* predictions (ITAG release 2.3 SL2.40) are used.

The exon-intron structure of all 47 *Sl*AQPs was analyzed using the tomato gene models (ITAG release 2.3 SL2.40) or by comparing experimentally determined EST sequences to the reference genome (Fig. 3). With some exceptions the number and the size of the exons (but not of the introns) is conserved within each AQP subfamily. This finding further validates the nomenclature proposed by our phylogenetic analysis (Fig. 1).

Most members of the *SlPIP* subfamily are characterized by four exons, the exceptions being *SlPIP2;1*, *SIPIP2;4* and *SlPIP2;6* which feature only three exons. The majority of the members of the *SlTIP* subfamily features three exons, while *SlTIP1;1* and *SlTIP1;3* lack the last intron. For *SlTIP1;3* no EST was available, so this finding could only be validated for *SlTIP1;1*. The genes assigned to the *SlNIP* subfamily mostly feature five exons. The exceptions were *SlNIP2;2* (four exons, no EST), *SlNIP4;3* (three exons, no EST) and *SlNIP5;1* (four exons confirmed by EST). The genes in the small subfamily of the *SlSIPs* seem to contain three exons. Only *SlSIP1;3* seemed to encode for a C-terminally truncated protein (two exons, no EST). The subfamily of *SlXIPs* was characterized by a conserved three-exon structure. Only *SlXIP1;6* deviated from that structure, featuring six predicted exons.

### Analysis of conserved, substrate determining amino acid residues

For the AQP family of transport proteins several conserved AA positions have been reported that influence substrate specificity by affecting pore diameter and hydrophobicity [38]–[40]. By careful visual inspection of AA sequence alignments of AQP subfamily members these position were detected (Table 2). Two highly conserved NPA motifs, found in loops B and E, were found to be critical for the transport function of AQPs [41]. In water-transporting AQPs, these NPA motifs together form a narrow pore, which aligns the transported water molecules into a single file [42]. However, also in some AQPs which were shown to transport substrates different from water two NPA motifs are found. Another set of four conserved residues forms the aromatic/Arginine filter (ar/R filter). The first two residues are located in helices 2 and 5 (H2 and H5), while the latter two are found in loop E (LE1 and LE2). It is suggested that these residues act as a size-exclusion barrier for substrate molecules [43]. In water-transporting AQPs these residues tend to be large and rather hydrophilic, as illustrated by the human AQP1 protein (F58-H182-C191-R197). In aquaglyceroproteins, residues forming the ar/R constriction are usually smaller and less hydrophilic (T48-G191-F200-R205 in human Glpf), allowing the transport of bulkier, more hydrophobic substances [38]. Finally, statistical analyses identified five key residues (named P1 to P5) that were proposed to discriminate between AQP- and GlpF-type AQPs [39]. The AA residues in these positions will be discussed for each subfamily. Also, when appropriate, potential phosphorylation sites or subfamily specific features will be discussed.

**Table 2:** Conserved specificity-determining residues in tomato aquaporins.

		NPA^1^		ar/R Filter				SDP^2^				
	Name	1st	2nd	H2	H5	LE1	LE2	P1	P2	P3	P4	P5
PIP	SlPIP1;1			F	H	T	R	M	S	A	F	W
	SlPIP1;2			F	H	T	R	Q	S	A	F	W
	SlPIP1;3			F	H	T	R	M	S	A	F	W
	SlPIP1;5			F	H	T	R	M	S	A	F	W
	SlPIP1;7			F	H	T	R	G	S	A	F	W
	SlPIP2;1			F	H	T	R	Q	S	A	F	W
	SlPIP2;4			F	H	T	R	Q	S	A	F	W
	SlPIP2;5			F	H	T	R	Q	S	A	F	W
	SlPIP2;6			F	H	T	R	Q	S	A	F	W
	SlPIP2;8			F	H	T	R	M	S	A	F	W
	SlPIP2;9			F	H	T	R	M	S	A	F	W
	SlPIP2;10			F	H	T	R	Q	S	A	F	W
	SlPIP2;11			F	H	T	R	M	S	A	F	W
	SlPIP2;12			F	H	T	R	Y	S	A	F	W
TIP	SlTIP1;1			H	I	A	V	T	S	S	Y	W
	SlTIP1;2			H	I	A	V	T	S	A	Y	W
	SlTIP1;3			H	I	A	V	T	S	A	Y	W
	SlTIP2;1			H	I	G	R	T	S	A	Y	W
	SlTIP2;2			H	I	G	R	T	S	A	Y	W
	SlTIP2;3			H	I	G	R	T	S	A	Y	W
	SlTIP2;5			H	I	G	R	T	S	A	Y	W
	SlTIP3;1			H	V	A	R	T	A	A	Y	W
	SlTIP3;2			H	T	A	R	T	A	A	Y	W
	SlTIP4;1			H	I	A	R	T	S	A	Y	W
	SlTIP5;1			N	V	G	Y	N	S	A	Y	W
NIP	SlNIP1;1	NPS		W	V	A	R	F	S	A	Y	L
	SlNIP1;2			W	V	A	R	F	S	A	Y	M
	SlNIP2;1			G	S	G	R	L	S	A	Y	I
	SlNIP2;2		NPT	-	S	G	R	F	S	A	Y	I
	SlNIP3;1			W	I	A	R	F	S	A	Y	I
	SlNIP3;2			W	V	A	R	F	S	A	F	V
	SlNIP4;1			W	V	A	R	F	S	A	Y	I
	SlNIP4;2			W	V	A	R	L	S	A	Y	I
	SlNIP4;3		-	W		-	-	L	-	-	-	
	SlNIP5;1	NPS	NPV	S	I	A	R	F	T	A	Y	L
	SlNIP6;1		NPV	T	I	A	R	L	T	A	Y	L
	SlNIP7;1			A	V	G	R	Y	S	A	Y	V
SIP	SlSIP1;1	NPT		V	T	P	N	C	A	A	Y	W
	SlSIP1;2			F	T	P	N	F	A	A	Y	W
	SlSIP1;3		-	F	-	-	-	F	-	-	-	-
	SlSIP2;1	NPL		F	K	G	S	I	V	A	Y	W
XIP	SlXIP1;1	NPV		I	T	A	R	V	C	P	F	W
	SlXIP1;2	NPI		I	T	A	R	V	C	P	F	W
	SlXIP1;3	NPI		I	T	A	R	V	C	P	F	W
	SlXIP1;4	NPV		A	T	A	R	V	C	P	F	W
	SlXIP1;5	NPV		I	T	V	R	V	C	P	F	W
	SlXIP1;6	SPV		I	T	A	R	V	C	A	F	W

1 Only non-standard NPA- motifs are shown.

2 Specificity determining positions according to Froger *et al.* 1998 [39].

### PIPs

All *Sl*PIPs featured the dual NPA motif characteristic for AQPs (Fig. S1). Also all *Sl*PIPs showed an ar/R filter configuration typical for a water-transporting AQP (F,H,T,R). In fact, these residues are identical to those found in the human AQP1, except for a C191T exchange. This seems to be a plant specific exchange, as it is also found in the PIPs from other plant species [17], [20], [19], [44]. The P1 position is more variable and filled by M/Q/G/Y, while the positions P2 to P5 are strictly conserved and filled with S-A-F-W. Member of the PIP subfamily in other plant species have been described to be positively regulated in their water transport activity through phosphorylation [45]–[48]. These phosphorylation sites were found to be conserved also in the *Sl*PIPs. More specifically, one S residue in loop B and E each was conserved in all *Sl*PIPs. Also multiple S residues at the C-terminus were present in most *Sl*PIP members while *Sl*PIP2;1 to *Sl*PIP2;10 featured a conserved S-X-R motif in their extreme C- terminus which is a recognition site for the protein kinase C [47], [49]. A number of other residues was found to be specific to either the *Sl*PIP1 or *Sl*PIP2 family members. Just before the second TMD a Q is found in *Sl*PIP1 proteins while a more hydrophobic L/V is found in *Sl*PIP2 proteins. In the fifth TMD L (PIP1s) is replaced by M (PIP2s) and after the sixth TMD a P (PIP1s) is replaced by A/M (PIP2s). Site-directed mutagenesis of PIP1 or PIP2 specific residues of radish AQPs established also an I (PIP1s) or V (PIP2s) located after the second NPA motif as critical for water transport activity [50]. Reciprocal mutations of these residues showed that a V in this position, as found in PIP2s, is increasing water transport activity compared to I. In tomato PIPs a V is found at this position in all *Sl*PIP2s and also *Sl*PIP1;7. This indicates that members of the *Sl*PIP2 subgroup might have water transport activity.

It is established that members of the PIP family function as water transporters enabling efficient transport of water into and out of the symplast (reviewed in [5], [7]). In addition to transporting water, PIP1 family member *Nt*AQP1 was reported to facilitate the diffusion of CO_2_ in the mesophyll [51], [52]. Using an *Arabidopsis PIP1;2* mutant it was shown that CO_2_ diffusion facilitated by PIP1;2 can become a limiting factor for photosynthesis [53]. It is also noteworthy that *At*PIP1;2 had almost no water transport activity. The structural basis for this specificity is currently not known. Given the high degree of conservation between tomato PIPs and functionally characterized PIPs from other plant species it is very likely that individual tomato PIPs also play a role in either water homeostasis or CO_2_ diffusion.

### TIPs

All *Sl*TIPs feature the two canonical NPA motifs (Fig. S2). The H2 residue of the ar/R filter region is H, except in *Sl*TIP5;1, where N is found. The H5 position is mostly I, except for *Sl*TIP3;1 (V), *Sl*TIP3;2 (T) and *Sl*TIP5;1 (V). The positions LE1 and LE2 were found to be specific for each subgroup in the *Sl*TIP subfamily. The *Sl*TIP1 subgroup is characterized by A (LE1) and an unusual V (LE2) instead of R, the *Sl*TIP2 subgroup by G (LE1) and R (LE2) and the TIP3 subgroup (and also *Sl*TIP4;1) by A (LE1) and R (LE2). As found for the other positions, TIP5;1 is deviating and showed G (LE1) and Y (LE2) residues. The position P1 in the *Sl*TIP subfamily was found to be a highly conserved T, except for *Sl*TIP5;1 (N). P2 was found to be S in all *Sl*TIPs but *Sl*TIP3;1 and *Sl*TIP3;2, where A is found in P2. P3 is occupied by A in almost all *Sl*TIPS, only *Sl*TIP1;1 had S substituted for A. P4 (Y) and P5 (W) were strictly conserved in all *Sl*TIPs.

In a previous study in tomato *Sl*TIP2;2 was shown to be a functional water transporter and overexpression in tomato resulted in improved fruit yield and plant biomass [27]. A number of reports (discussed in Hove *et al.*, 2011 [38], and references therein) on other plant species characterized members of the TIP subfamily also as transporters of small solutes such as NH_4_^+^ (*At*TIP2;1 and 2;3, *Ta*TIP2;1 and 2;2) [54]–[57], H_2_O_2_ (*At*TIP1;1, 1;2 and 2;3) [58]–[60] and urea (*At*TIP1;1 to 1;3, 2;1, 5;1 and *Nt*TIP4;1) [61]–[64]. Since the residues forming the central pore and determining the specificity (NPA motifs, ar/R filter, P1 to P5) are conserved across species in these subgroups, there is a possibility that also the tomato TIPs will be able to transport solutes. As in other species (*Arabidopsis*, rice, soybean), also in tomato one unusual member of the TIP family was found (*Sl*TIP5;1). The AA sequence of *Sl*TIP5;1 is less similar to a hypothetical *Sl*TIP consensus sequence compared to the other *Sl*TIP family members, resulting in *Sl*TIP5;1 forming a single-gene clade within the *Sl*TIP subfamily. Recently it was found that in *Arabidopsis TIP5;1* is highly expressed in pollen and transports water and urea [65]. Also, expression of *AtTIP5;1* was shown to be induced under elevated B conditions and overexpression of *AtTIP5;1* enhanced the tolerance to high B conditions [66]. This tissue and stimulus-specific expression might be one reason, why no EST of *Sl*TIP5;1 was found in the databases.

### NIPs

In the *SlNIP* subfamily the NPA motifs showed some variability (Fig. S3). In *Sl*NIP1;1 and *Sl*NIP5;1 the first NPA motif is changed to NPS, while in *Sl*NIP2;2 *Sl*NIP5;1 and *Sl*NIP6;1 the second NPA motif is changed to NPT (*Sl*NIP2;2) or NPV (*Sl*NIP5;1, *Sl*NIP6;1). Also the residues that form the ar/R constriction were more variable. However, within the different subgroups a higher degree of conservation was detected. The ar/R filter in the *Sl*NIP1, *Sl*NIP3 and *Sl*NIP4 subgroup consisted of W (H2), V/I (H5), A (LE1) and R (LE2). *Sl*NIP*4;3* was found to encoded a C-terminally shortened protein, compared to the rest of the *Sl*NIP subfamily, so only H2 could be specified. In the *Sl*NIP2 subgroup the ar/R filter consisted of G (H2), S (H5), G (LE1) and R (LE2), although a deletion in the second transmembrane domain of *Sl*NIP2;2 made it impossible to specify H2 in this protein. The positions P1 to P4 were mostly conserved in the *Sl*NIP subfamily, the consensus sequence being F/L (P1) S (P2), A (P3) and Y (P4). P5 was found to be more variable showing L, M, I and V residues.

The *Sl*NIP subfamily is named after its first described member, soybean nodulin 26 (reviewed in [67]), which is found in the symbiosome membrane of the nitrogen-assimilating root nodules. It was found to transport water (albeit with a lower conductivity than true AQPs) and also solutes like formamide, glycerol [68], [69] and ammonia [70]. The *Sl*NIP subgroups *Sl*NIP1, *Sl*NIP3 and *Sl*NIP4 show an ar/R filter configuration consistent with that of soybean Nodulin 26, indicating water- as well as solute-transport capability [71], [72]. In cereals members of the NIP2 subgroup were characterized as Si transporter [73]–[75]. Whereas the ar/R filter positions and the P1 to P5 positions are almost perfectly conserved compared to barley, maize and rice in *Sl*NIP2;1, *Sl*NIP2;2 lacks position H2 since a 17 AA stretch from TMD2 is missing. Also no EST evidence for *Sl*NIP2;2 was found. While *Sl*NIP2;1 might be a functional Si transporter, functionality of *Sl*NIP2;2 is questionable. For the *Arabidopsis* orthologs of *Sl*NIP5;1, 6;1 and 7;1 it was shown that they play a role in B homeostasis in the shoot and probably in the anther [76]–[78]. Orthologs from both organisms share non-canonical NPA-motifs and also the ar/R filter region was found to be conserved between organisms. This indicates that the *Sl*NIPs 5;1, 6;1 and 7;1 are B transporters, however experimental evidence is needed to confirm this. Nodulin 26, the founding member of the NIP subfamily was shown to be phosphorylated by the CDPK (calcium dependent protein kinase) at an S residue in the C-terminal region which enhanced water permeability [79], [80]. Recognition sites for CDPK phosphorylation are also found in the C-terminus of *Sl*NIP1 and *Sl*NIP4 members (except *Sl*NIP4;1), implying regulation by phosphorylation (Fig. S3).

### SIPs

The *Sl*SIP subfamily has a less conserved first NPA motif, while the second NPA motif is perfectly conserved in all full-length members (Fig. S4). Position H2 of the ar/R filter is occupied by a hydrophobic and aromatic V or F. The positions H5 and LE1 are filled by the more polar AA T and P in *Sl*SIP1;1 and 1;2. In *Sl*TIP2;1 the unique combination of K (H5) and G (LE1) is found. Position LE2 has a unique N or S residue in place of the expected R. The position P1 to P5 of the SIP1 subgroup were C/F, A, A, Y and W, while in *Sl*SIP2;1 I, V, A, Y, W were found. *SlSIP1;3* was found to encode a C-terminally truncated protein compared to the rest of the family. Since also no EST evidence could be detected, it likely represent a pseudogene. All full-length *Sl*SIPs contained several K residues in their C-terminal region, which is characteristic for members of the SIP family [10] (Fig. S4). Members of the *Sl*SIP1 subgroup were shown to transport water and localize to the ER membrane *in vitro* [36]. The subcellular localization of the *Sl*SIPs however was predicted to be the tonoplast. So far no data regarding the physiological role of SIPs is available.

### XIPs

All members of the *SlXIP* subfamily showed a modified first NPA motif (N/S, P, V/I), whereas the second NPA motif is extended to an NPARC motif, reported to be conserved in XIP subfamily members from other plant [12] (Fig. S5). The ar/R filter is comprised of I/A (H2), T (H5), A/V (LE1) and R (LE2). Since the first three AA of the ar/R filter have rather hydrophobic residues, the *SlXIPs* might be involved in transport of molecules other than water [38]. The positions P1 to P5 are occupied with V, C, P/A, F and W conserved in all members of the *Sl*XIP subfamily. The XIP1 paralogues from several *Solanaceae* species, including tomato, tobacco and potato were recently characterized [11]. In these experiments XIPs showed reduce water transport activity compared to AQPs from the PIP subfamily while being able to transport substrates like urea, H_2_O_2_ and B when expressed in a yeast system. Furthermore, the proteins were localized to the PM of epidermal and parenchyma cells. Since the additional XIPs discovered in tomato showed mostly conserved ar/R filter regions it is very likely that they also function as solute transporters, although their physiological substrates are still unknown.

### Expression analysis

**Figure 4:**
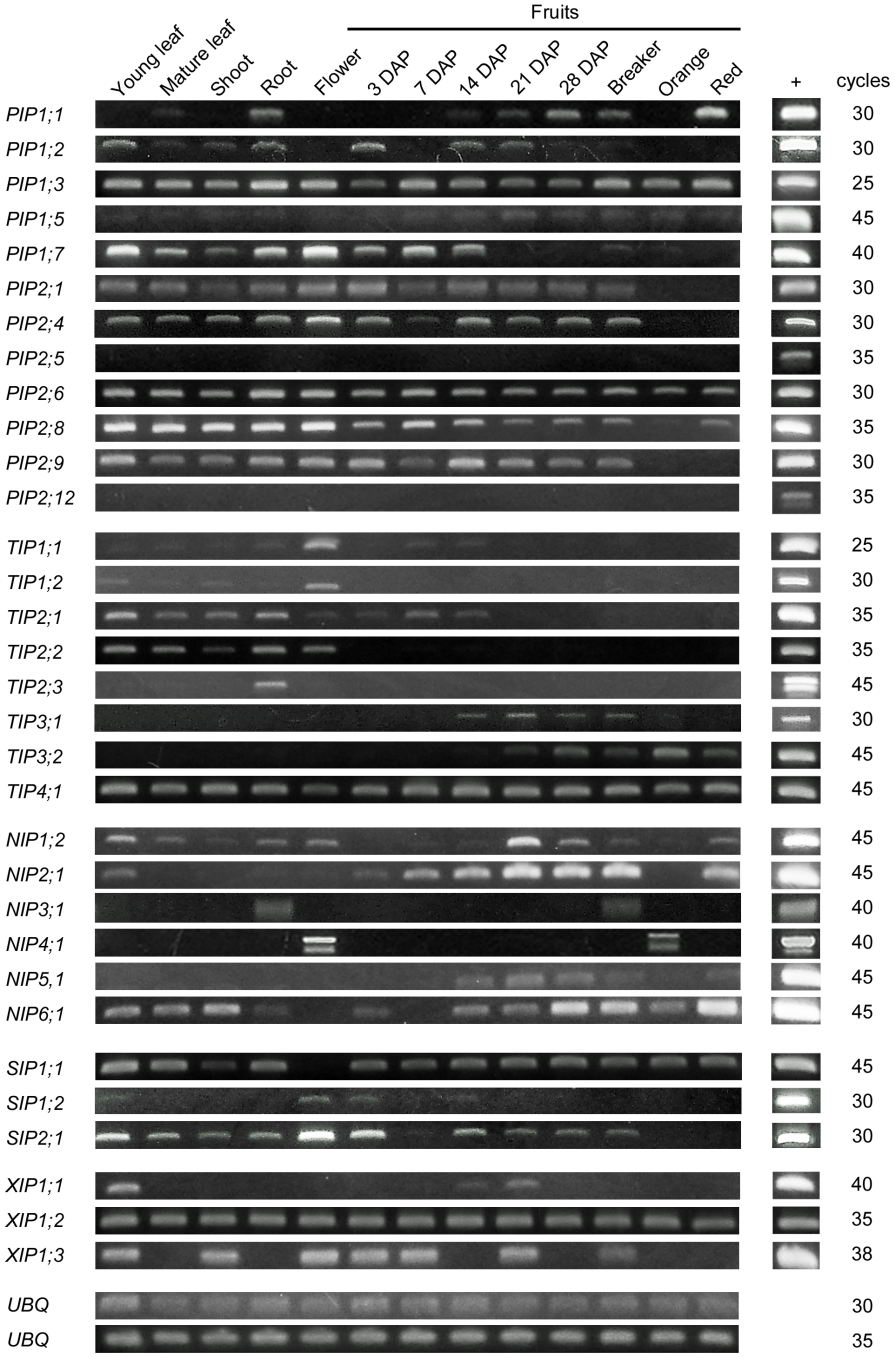
Expression analysis of selected tomato aquaporins. Shown is a semi-quantitative RT-PCR analysis of tomato aquaporins. RNA was extracted from the indicated tissues, transcribed to cDNA and used as a template for PCR. + indicates reactions using the respective EST-containing plasmid DNA as a template. Gene-specific primers (amplicons *ca.* 200 bp) were used to analyze expression levels by PCR. UBQ indicates a tomato ubiquitin gene used as a constitutively expressed control gene. DAP = days after pollination. Results are representative of two technical replicates for each tissue.

The expression of 32 tomato AQPs in different vegetative tissues and in developing fruits of the tomato cultivar ‘Micro-Tom’ was analyzed by semi-quantitative RT-PCR (Fig. 4). Only AQPs that were represented by at least one EST in the analyzed tissues were included in the analysis. For most of the analyzed AQPs expression in at least one tissue could be detected. No expression could be detected in any tissue for *SlPIP2;5* and *SlPIP2;12*. There is the possibility that these genes are only expressed at a detectable level after exposure to a specific stimulus. Several genes (*SlPIP1;3*, *SlPIP2;1*, *SlPIP2;4*, *SlPIP2;6*, *SlPIP2;8*, *SlPIP2;9*, *SlTIP4;1*, *SlSIP1;1*, *SlXIP1;2*) seemed to be expressed in all analyzed tissues, indicating a role in constitutive transport processes throughout the plant. A strong signal in cDNA from root tissue, but not from shoot or leaf tissues, was obtained for *Sl*PIP1;1, *Sl*TIP2;3 and *Sl*NIP3;1 indicating a specific function in roots. Based on the known properties, two functions for AQPs in roots seem likely. First, water uptake and conductance in roots is, at least in parts controlled by AQPs [81]. Roots are also the primary uptake organ for macro- and micronutrients. It is conceivable that AQPs play a role in the uptake and translocation of nutrients, illustrated by the effect of *At*TIP5;1 on B homeostasis [66].

Several *SlAQPs* were found to be expressed in dynamic, fruit-specific pattern, indicating a role in fruit development, most likely transport of water or solutes. Increasing amounts of *SlNIP2;1* and *SlNIP6;1* transcripts could be detected in flowers and fruits from the earliest (3 days after pollination, 3 DAP) to the last stage of fruit development (Red). Expression of *SlPIP1;1* and *SlTIP3;2* started at 14 DAP and increased with proceeding fruit development. *SlTIP3;1*, *SlNIP5;1*, *SlXIP1;1* transcripts were found exclusively in fruits during mid-development (around 21 DAP). *SlPIP1;2*, *SlPIP1;7* and *SlSIP2;1* expression was strongest in early-to-mid fruit development but ceased during the later stages. Expression of *SlNIP4;1* was restricted to the flower and the ‘Orange’ stage of fruit development. Developing fruits are strong sink organs and the accumulation of sugars in them causes a negative water potential. It seems likely that at least some AQPs identified here as expressed in fruits are necessary for water accumulation during fruit development. It can be speculated that AQPs also facilitate water movement within the fruit between apoplast and symplast and on the intercellular level between the cytosol and the vacuole. The expression analysis clearly identified several tomato AQPs expressed in a tissue- or development-specific manner. Further functional analyses of AQPs, selected on the basis of these data, are now necessary to understand the roles of individual AQP members in their respective tissues.

## Conclusion

In this study a comprehensive overview of the AQP family in tomato is presented. Comparable to other plant species, the AQP family consists of 47 highly similar members, which can be assigned to five phylogenetic subfamilies. In-detail sequence comparisons and expression analysis allows us to speculate on the contribution of single AQP members to water or solute homeostasis in tomato. Aside from being of commercial value, tomato is also a model crop for fleshy fruit development. The role of AQPs during fleshy fruit development is still unknown. It is presumed that water movement into the developing fruit is at least partially mediated by AQPs. By genome-wide identification of tomato AQPs and measuring expression levels during fruit development we did a first step towards identifying AQPs responsible for water transport into developing tomato fruits. Now experiments designed to test the physiological functions of AQPs can be performed on the basis of these data to elucidate the role of selected AQPs during fruit development. Since efficient transformation protocols exist for tomato it should be possible to analyze the function of selected genes by creating transgenic knockdown or overexpressing plants. Also localization of AQP expression on the tissue level and analyses of the subcellular localizations of AQP proteins will aid in defining a function for single AQPs.

## Supporting Information

Figure S1 **Alignment of AA sequences of** ***Sl*** **PIP subfamily members.** Shown is an AA sequence alignment of all *Sl*PIPs. Black lines above the alignment indicate predicted transmembrane domains. The two conserved NPA motifs are shown in bold letters. Residues comprising the ar/R filter are marked in grey and labelled H2, H5, LE1 and LE2. Residues occupying conserved positions one to five (from N- to C-terminus: P1 to P5) are marked in yellow. Columns or regions with conserved putative phosphorylation sites are marked by an asterisk. An S-X-A motif for putative phosphorylation by PKC is marked in blue. Note that for *Sl*PIP2;12′ the deduced AA sequence from the a corrected EST is shown (see main text).
(DOCX)

Figure S2 **Alignment of AA sequences of** ***Sl*** **TIP subfamily members.** Shown is an AA sequence alignment of all *Sl*TIPs. Black lines above the alignment indicate predicted transmembrane domains. The two conserved NPA motifs are shown in bold letters Residues comprising the ar/R filter are marked in grey and labelled H2, H5, LE1 and LE2. Residues occupying conserved positions one to five (from N- to C-terminus P1 to P5) are marked in yellow.
(DOCX)

Figure S3 **Alignment of AA sequences of** ***Sl*** **NIP subfamily members.** Shown is an AA sequence alignment of all *Sl*NIPs. Black lines above the alignment indicate predicted transmembrane domains. The two conserved NPA motifs are shown in bold letters. Residues comprising the ar/R filter are marked in grey and labelled H2, H5, LE1 and LE2. Residues occupying conserved positions one to five (from N- to C-terminus P1 to P5) are marked in yellow. A conserved Calcium-dependent protein kinase recognition site in the C-terminus is marked with blue boxes.
(DOCX)

Figure S4 **Alignment of AA sequences of** ***Sl*** **SIP subfamily members.** Shown is an AA sequence alignment of all *Sl*SIPs. The two conserved NPA motifs are shown in bold letters. Residues comprising the ar/R filter are marked in grey and labelled H2, H5, LE1 and LE2. Residues occupying conserved positions one to five (from N- to C-terminus P1 to P5) are marked in yellow.
(DOCX)

Figure S5 **Alignment of AA sequences of** ***Sl*** **XIP subfamily members.** Shown is an AA sequence alignment of all *Sl*XIPs. The two conserved NPA motifs are shown in bold letters. Residues comprising the ar/R filter are marked in grey and labelled H2, H5, LE1 and LE2. Residues occupying conserved positions one to five (from N- to C-terminus P1 to P5) are marked in yellow. Note that for *Sl*XIP1;2′ the deduced AA sequence from a corrected EST is shown (see main text).
(DOCX)

Figure S6 **Phylogenetic analysis of aquaporins from tomato and 13 other species.** Shown is a phylogenetic tree from an alignment of AA sequences from all identified MIPs from *Solanum lycopersicum* together with MIPs from *Arabidopsis thaliana* and *Oryza sativa*. For the XIP subfamily sequences from *Physcomitrella patens*, *Populus trichocarpa*, *Ricinus communis*, *Gossypium hirsutum*, *Gossypium raimondii*, *Lactuca scariola*, *Citrus clementine*, *Citrus sinensis*, *Ipomoea nil*, *Solanum tuberosum* and *Nicotiana tabacum* were used. For tomato the gene name and the best hit EST are given. If no EST was found the locus is given. For Arabidopsis and rice the gene name and the locus are given; for other species the NCBI accession number or the JGI protein ID is given. Bold font indicates tomato MIPs. #^1^ indicates EST is not full length. #^2^ indicates EST contained a frameshift leading to premature termination; Putative full-length AA sequence was used.
(DOCX)

Table S1 **Sequences of oligonucleotides and PCR program settings used for gene expression analysis.** Shown are the sequences of the forward (FWD) and the (REV) primer used to analyze the expression of each *Sl*AQP. Below each primer pair the PCR program used for each target gene is given.
(DOCX)
