# Supplementary material for: Genome-Wide Identification and Expression Analysis of Aquaporins in Tomato
Source: PLoS One. 2013 Nov 19;8(11):e79052. doi: 10.1371/journal.pone.0079052 (PMC3834038; doi:10.1371/journal.pone.0079052)
Supplement: Figure S1 — Alignment of AA sequences of Sl PIP subfamily members. Shown is an AA sequence alignment of all SlPIPs. Black lines above the alignment indicate predicted transmembrane domains. The two conserved NPA motifs are shown in bold letters. Residues comprising the ar/R filter are marked in grey and labelled H2, H5, LE1 and LE2. Residues occupying conserved positions one to five (from N- to C-terminus: P1 to P5) are marked in yellow. Columns or regions with conserved putative phosphorylation sites are marked by an asterisk. An S-X-A motif for putative phosphorylation by PKC is marked in blue. Note that for SlPIP2;12′ the deduced AA sequence from the a corrected EST is shown (see main text). (DOCX) [file pone.0079052.s001.docx]

*Sl*PIP1;1 1 ---MAENKEEDVNLGANKFREPQPLG--------TSAQT-DKDYKEPPPAPLYEPGELSS
*Sl*PIP1;2 1 ----MEGKEEDVKVGANKYSERQPLG--------TSAQS--KDYKEAPPAPLFEAGELHS
*Sl*PIP1;3 1 ---MAENKEEDVNLGANKYRETQPLG--------TAAQT-DKDYKEPPPAPLFEPGELSS
*Sl*PIP1;5 1 ---MAENKEEDVKLGANKFRETQPLG--------TAAQT-DKDYKEPPPAPLFEPGELSS
*Sl*PIP1;7 1 ----MEHREEDVRLGANKYSERQAIG--------IAAQSEDKDYKEPPPAPLFEPGELMS
*Sl*PIP2;1 1 -----MAKDMEVGT------EYAP-----------------KDYQDPPPAPLIDPEELGK
*Sl*PIP2;4 1 -----MTKEVT-------------------------DFS-AKDYTDPPPAPLVDFEELRQ
*Sl*PIP2;5 1 -----MAKDMEY--GN---DQYAP----------------SKDYQDPPPAPLIDPEELGK
*Sl*PIP2;6 1 -----MTKEVE---AA--HE-QA-V-----------EYS-AKDYTDPPPAPLIDFEELTK
*Sl*PIP2;8 1 -----MSKEVIEE--GQVQQH-------------------GKDYVDPPPAPLLDFAELKL
*Sl*PIP2;9 1 -----MSKDVIEE--GQAHHH-------------------GKDYVDPPPAPLLDMAELTK
*Sl*PIP2;10 1 MVRHEWLKESTRAKK-QRQPKKKTETQKWAKIWRLVLNMHQKTYQDPPPAPLIDPEELGK
*Sl*PIP2;11 1 ---------------------------------------MVKDYVDPPSAPLFQTAELYN
*Sl*PIP2;12’ 1 -----MSNEVSSALP---ERSSSP----------------AKDYHEPPPAPFIGAAELKK

 H2
*Sl*PIP1;1 49 WSFYRAGIAEFMATFLFLYITILTVMGLKRSDSL------CSSVGIQGVAWAFGGMIFAL
*Sl*PIP1;2 47 WSFWRAGIAEFMATFLFLYITVLTVMGYSRANSK------CSTVGVQGIAWAFGGMIFAL
*Sl*PIP1;3 49 WSFYRAGIAEFMATFLFLYITILTVMGLKRSDSL------CSSVGVQGVAWAFGGMIFAL
*Sl*PIP1;5 49 WSFYRAGIAEFMATFLFLYITILTVMGLKRSDSL------CSSVGIQGVAWAFGGMIFAL
*Sl*PIP1;7 49 WSFYRAGIAEFVATFLFLYITVLTVMGVSKSDSK------CSTVGIQGIAWAFGGMIFAL
*Sl*PIP2;1 33 WSFYRAIIAEFIATLLFLYITVLTVIGYKSQSSTDQ----CGGVGILGIAWAFGGMIFVL
*Sl*PIP2;4 30 WSFYRAIIAEFIATLLFLYVTILTVIGYKHQADVDAGGDVCGGVGILGIAWAFGGMIFIL
*Sl*PIP2;5 35 WSFYRAIIAEFIATLLFLYITVLTVIGYKSQSDGDQ----CGGVGILGIAWAFGGMIFVL
*Sl*PIP2;6 37 W*SL*YRAAIAEFIATLLFLYITILTVIGYKHQADVKAGGDICGGVGLLGIAWAFGGMIFVL
*Sl*PIP2;8 35 WSFYRALIAEFIATLLFLYVTVATVIGHKKLNGADK----CDGVGILGIAWAFGGMIFVL
*Sl*PIP2;9 35 WSFYRALIAEFIATLLFLYVTVATVIGHKKLNALDQ----CDGVGILGIAWAFGGMIFVL
*Sl*PIP2;10 60 WSFYRAIIAEFIATLLFLYITVLTVIGYKSQSSTDQ----CGGVGILGIAWAFGGMIFVL
*Sl*PIP2;11 22 WSFYRALIAEFVATLLFLYVSVATVIGHKKQLGP------CDGVGLVGIAWAFGGMIFVL
*Sl*PIP2;12’ 37 WALYRALIAEFVATLLLLYIGQLTIMGYKSESDHDP----CGSVGLLGVAWVFGGMVFIL


*Sl*PIP1;1 103 VYCTAGISGGHI**NPA**VTFGLFLARKLSLTRAVFYMVMQCLGAICGAGVVKGFMQGPYQRL
*Sl*PIP1;2 101 VYCTAGISGGHI**NPA**VTFGLFLARKLSLTRAVFYIVMQCLGAICGAGVVKGFQPSLFETK
*Sl*PIP1;3 103 VYCTAGISGGHI**NPA**VTFGLFLARKLSLTRAVFYIVMQCLGAICGAGVVKGFMVGPYERL
*Sl*PIP1;5 103 VYCTAGISGGHI**NPA**VTFGLFLARKLSLTRAVFYMVMQCLGAICGAGVVKGFMVGPYQRL
*Sl*PIP1;7 103 VYCTAGISGGHI**NPA**VTFGLFLARKLSLTRAVFYMVMQCLGAICGAALVKAFGKTLYQTK
*Sl*PIP2;1 89 VYCTAGISGGHI**NPA**VTFGLFLARKVSLVRAIMYIVAQCLGAICGCGLVKAFQKAYYVKY
*Sl*PIP2;4 90 VYCTAGISGGHI**NPA**VTFGLFLARKVSLIRAVLYMVAQCLGAICGVGFVKAFQSAYYNRY
*Sl*PIP2;5 91 VYCTAGISGGHI**NPA**VTFGLLLARKVSLVRAIFYIVAQCLGAICGCGLVKLFQKAYYVKY
*Sl*PIP2;6 97 VYCTAGISGGHI**NPA**VTFGLFLARKVSLIRAILYMVAQCLGAICGVGFVKAFQSAYYNRY
*Sl*PIP2;8 91 VYCTAGISGGHI**NPA**VTFGLFLARKVSLVRAVGYIIAQCLGAICGVGFVKAFMTHPYNAL
*Sl*PIP2;9 91 VYCTAGISGGHI**NPA**VTFGLFLARKVSLIRAVAYIIAQSLGAICGVGFVKAFMKHYYNTE
*Sl*PIP2;10 116 VYCTAGISGGHI**NPA**VTFGLFLARKVSLVRAIMYIVAQCLGAICGCGLVKAFQKAYYVKY
*Sl*PIP2;11 76 VYSTAGISGGHI**NPA**VTFGLLLARKVSLLRAVAYMVAQCLGAICGVGLVKGVMKDDYTKH
*Sl*PIP2;12’ 93 VYCTAGISGGHI**NPA**VTFGLFLSRKISLIRGLLYIVVQYLGAICGTALVKAIYKSKFELY


*Sl*PIP1;1 163 GGGANVVQPGYTKGDGLGAEIIGTFVLVYTVFSATDAKRNARDSHVPILAPLPIGFAVFL
*Sl*PIP1;2 161 GGGANVVAHGYTKGDGLGAEIIGTFVLVYTVFSATDAKRNARDSHVPILAPLPIGFAVFL
*Sl*PIP1;3 163 GGGANVVNPGYTKGDGLGAEIIGTFVLVYTVFSATDAKRSARDSHVPILAPLPIGFAVFL
*Sl*PIP1;5 163 GGGANVVNPGYTKGDGLGAEIIGTFVLVYTVFSATDAKRNARDSHVPILAPLPIGFAVFL
*Sl*PIP1;7 163 GGGANVVNVGYTKGDGLGAEIIGTFVLVYTVFSATDAKRSARDSHVPILAPLPIGFAVFL
*Sl*PIP2;1 149 GGGANTLNDGYSTGTGLGAEIIGTFVLVYTVFAATDPKRNARDSHVPVLAPLPIGFAVFM
*Sl*PIP2;4 150 GGGVNVMAGGHSKGVGLGAEIIGTFVLVYTVFSATDPKRNARDSHVPVLAPLPIGFAVFM
*Sl*PIP2;5 151 GGGANELAVGYNIATGLGAEIIGTFVLVYTVFSATDPKRNARDSHVPVLAPLPIGFAVFM
*Sl*PIP2;6 157 GGGVNVMAGGHTKGVGLAAEIIGTFVLVYVVFSATDPKRSARDSHVPVLAPLPIGFAVFM
*Sl*PIP2;8 151 GGGANFVQSGYNNGTALGAEIIGTFVLVYTVFSATDPKRSARDSHIPVLAPLPIGFAVFM
*Sl*PIP2;9 151 GGGANFVQPGYNNGTALGAEIIGTFVLVYTVFSATDPKRSARDSHVPVLAPLPIGFAVFM
*Sl*PIP2;10 176 GGGANTLNDGYSTGTGLGAEIIGTFVLVYTVFAATDPKRNARDSHVPVLAPLPIGFAVFM
*Sl*PIP2;11 136 GGGANTVAVGYSTGAALGAEIIATFLLMYTVFSATDAKRKARDSHVPVLAPLPIGFSVFM
*Sl*PIP2;12’ 153 GGGVNSVSPGYTRGVAWSAEMIGTFVLVYTVLSATDSKRNARDSHVPVLAPLPIGFAVFL

*

*

*

*

H5 LE1 LE2

*Sl*PIP1;1 223 VHLATIPITGTGI**NPA**RSNGAAIIFNQDQAWDDHWIFWFGNFIGAALAAIYHQIIIRAIP
*Sl*PIP1;2 221 VHLATIPITGTGI**NPA**RSLGAAIVYNKEHAWDDHWIFWVGPFIGAALAALYHQVIIRAIP
*Sl*PIP1;3 223 VHLATIPITGTGI**NPA**RSLGAAIIFNKDEAWDDHWIFWVGPFIGAALAAVYHQIIIRAIP
*Sl*PIP1;5 223 VHLATIPITGTGI**NPA**RSLGAAIIYNDEHAWNDHWIFWVGPMIGAALAAIYHQIIIRAMP
*Sl*PIP1;7 223 VHLATIPVPGTGI**NPA**RSLGAAVIYNNEQAWKDPWIFWVGPFIGAALGALYPQVVIRAIP
*Sl*PIP2;1 209 VHLATIPVTGTGI**NPA**RSFGAAVVYGHNKAWDDQWIFWVGPFIGAAIAAFYHQFILRAGA
*Sl*PIP2;4 210 VHLATIPVTGTGI**NPA**RSFGAAVIFNGDKAWDEHWIFWVGPFIGAFIAAFYHQFVLRAGA
*Sl*PIP2;5 211 VHLATIPITGTGI**NPA**RSFGAAVIYGKNKSWDDQWIFWVGPFIGAAIAAIYHQYILRAGA
*Sl*PIP2;6 217 VHLATIPITGTGI**NPA**RSFGAAVIFNGDKAWDDHWIFWVGPFFGAFIAAVYPQYIFRAGA
*Sl*PIP2;8 211 VHLATIPITGTGI**NPA**RSFGAAVIADNKNVWDDQWIFWVGPFVGALLAAAYHQYILRAAA
*Sl*PIP2;9 211 VHLATIPITGTGI**NPA**RSFGAAVIYGNEKIWDDQWIFWVGPMVGAMAAAIYHQFILRAGA
*Sl*PIP2;10 236 VHLATIPVTGTGI**NPA**RSFGAAVVYGHNKAWDDQWIFWVGPFIGAAIAAFYHQFILRAGA
*Sl*PIP2;11 196 VHLATIPITGTGI**NPA**RSFGAAVIYNDTTAWNDHWIFWVGPFLGALAAVIYHQQILRGHA
*Sl*PIP2;12’ 213 VHLATIPITGTGI**NPA**RSLGAAVIYNQQIAWEDNWDLFWRTFHRSTYCSNLPTNTEGMQM


*Sl*PIP1;1 283 FKSRA------------
*Sl*PIP1;2 281 FKSGN------------
*Sl*PIP1;3 283 FKSSRS-----------
*Sl*PIP1;5 283 FHRS-------------
*Sl*PIP1;7 283 FKSK-------------
*Sl*PIP2;1 269 VKALGSFRSNA------
*Sl*PIP2;4 270 IKALGSFRSTA------
*Sl*PIP2;5 271 SKSINSFRSNA------
*Sl*PIP2;6 277 IKALGSFRSNA------
*Sl*PIP2;8 271 IKALGSFRSNATN----
*Sl*PIP2;9 271 VKALGSFRSNQTN----
*Sl*PIP2;10 296 VKALGSFRSNA------
*Sl*PIP2;11 256 AKAF-------------
*Sl*PIP2;12’ 273 EISDATYICSRICVCDL


Figure S1: Alignment of AA sequences of SlPIP subfamily members.

*

Shown is an AA sequence alignment of all *Sl*PIPs. Black lines above the alignment indicate predicted transmembrane domains. The two conserved NPA motifs are shown in bold letters. Residues comprising the ar/R filter are marked in grey and labelled H2, H5, LE1 and LE2. Residues occupying conserved positions one to five (from N- to C-terminus: P1 to P5) are marked in yellow. Columns or regions with conserved putative phosphorylation sites are marked by an asterisk. An S-X-A motif for putative phosphorylation by PKC is marked in blue. Note that for *Sl*PIP2;12’ the deduced AA sequence from the a corrected EST is shown (see main text).
