# Supplementary material for: Genome-Wide Identification and Expression Analysis of Aquaporins in Tomato
Source: PLoS One. 2013 Nov 19;8(11):e79052. doi: 10.1371/journal.pone.0079052 (PMC3834038; doi:10.1371/journal.pone.0079052)
Supplement: Figure S2 — Alignment of AA sequences of Sl TIP subfamily members. Shown is an AA sequence alignment of all SlTIPs. Black lines above the alignment indicate predicted transmembrane domains. The two conserved NPA motifs are shown in bold letters Residues comprising the ar/R filter are marked in grey and labelled H2, H5, LE1 and LE2. Residues occupying conserved positions one to five (from N- to C-terminus P1 to P5) are marked in yellow. (DOCX) [file pone.0079052.s002.docx]

*Sl*TIP1;1 1 ----------------------MPINQITIGSHEELRHPGALKAALAEFISTLIFVFAGQ
*Sl*TIP1;2 1 ----------------------MPISRIAIGNLAEATKPDALKAATAEFFSMLIFVFAGS
*Sl*TIP1;3 1 ----------------------MPISRIAIGRREEATHPDALKAALAEFISTLIFVFAGS
*Sl*TIP2;1 1 ------------------------MPCIAFGRFDDSFSLGSIKAYIAEFISTLLFVFAGV
*Sl*TIP2;2 1 -----------------------MAGGVAIGSFSDSFSVVSLKSYLAEFISTLIFVFAGV
*Sl*TIP2;3 1 ------------------------MVKIAFGSIGDSLSVGSLKAYLAEFIATLLFVFAGV
*Sl*TIP2;5 1 MGSSPFKQLTQPRNKHIANYSFSQNALYSLGRFDDSLNCGSIKAYLAEFISTLLFVFAGV
*Sl*TIP3;1 1 ----------------------MQPRRYEFGRADEATHPDSVRATLSEFLSTFIFVFAGE
*Sl*TIP3;2 1 --------------------MAMPARRYAFGRADEATHPDSMRATLSELLSTFIFVFAGE
*Sl*TIP4;1 1 ------------------------MAKIAVGSSREAIQPDCIQALIVEFICTFLFVFAGV
*Sl*TIP5;1 1 ------------------------MASLAS-RLQHSVTPNALRSYLAEFLSTFFFVFAAA

 H2
*Sl*TIP1;1 39 GSGMAFNKLTDG-VATPAGLISASIAHAFGLFVAVSVGANISGGHV**NPA**VTFGAFVGGNI
*Sl*TIP1;2 39 GSGMAFGKLTNGGAATPAGLISASIAHAFALFVAVSVGANISGGHV**NPA**VTFGAFVGGHI
*Sl*TIP1;3 39 GSGVAFSKLTGGGANTPTGLIAAAIAHAFGLFVAVSVGANISGGHV**NPA**VTFGAFVGGNI
*Sl*TIP2;1 37 GSAIAYNKVTADAALDPSGLVAVAVCHGFALFVAVAIAANISGGHV**NPA**VTFGLALGGQI
*Sl*TIP2;2 38 GSAIAYGKLTTNAALDPAGLVAIAVCHGFALFVAVSISANISGGHV**NPA**VTCGLTFGGHI
*Sl*TIP2;3 37 GSAIAFNKLTSGAALDPAGLVAIAVAHAFALFVGVSMAANISGGHL**NPA**VTLGLAVGGNI
*Sl*TIP2;5 61 GSAIAYNKLTADAALDPAGLVAVAVCHGFALFVAVSIGANISGGHV**NPA**VTFGLALGGQI
*Sl*TIP3;1 39 GSVLALDKLYPDRALGASRLTAIALAHAFSLFAAVASSMNVSGGHI**NPA**VTFGALVGGRV
*Sl*TIP3;2 41 GSVLAIDKLYPDTGLGSSRLIVIALAHAFSFFAAVASSLNVSGGHI**NPA**VTFGSLVGGRI
*Sl*TIP4;1 37 GSAMAANKLNGD---PLVSLFFVAMAHALVVAVTISAGFRISGGHL**NPA**VTLGLCMGGHI
*Sl*TIP5;1 36 GASMSTRKMVPDATSDPSSLVAIAVANAFALSVAVYISANISGGHV**NPA**VTFGMAVGGHI


*Sl*TIP1;1 98 TLFRGILYIIAQLLGSTAACALLEFATGGMSTGSFALSAGVSVWNAFVFEIVMTFGLVYT
*Sl*TIP1;2 99 TLFRSVLYWIAQLLGSVVACVLLKFSTGGLETSAFALSSGVTPWNAVVFEIVMTFGLVYT
*Sl*TIP1;3 99 TLLRGILYWIAQLLGSVVACLLLKFTTGGMEIGAFSLSNGVGVGNALVLEIVMTFGLVYT
*Sl*TIP2;1 97 TLLTGLFYWIAQLLGAIVGCYLLKVVTGGMAVPIHGVAAGVGAAEGVVMEIIITFALVYT
*Sl*TIP2;2 98 TFITGSFYMLAQLTGAAVACFLLKFVTGGCAIPTHGVGAGVSILEGLVMEIIITFGLVYT
*Sl*TIP2;3 97 TILTGLFYWVAQLLGSTVACLLLKYVTNGLAVPTHGVAAGMSGAEGVVMEIVITFALVYT
*Sl*TIP2;5 121 TLLTGLFYWVAQLLGAIVACCLLKFVTGGLTVPIHGVAAGVGATEGVVMEIIITFALVYT
*Sl*TIP3;1 99 SVLRAMYYWIGQLLGAVVASALLRLATDGLRPVGFAVASGVGNGNALVMEIVMTFGLVYT
*Sl*TIP3;2 101 SVVRAIYYWVAQLFGSVLASLLLRLATDGLRPRGFSVAAGVGNLNALVMEIVMTFGLMYT
*Sl*TIP4;1 94 TVFRSILYWIDQLLASVAACALLNYLTDGMITPVHTLANGMSYWQGLIMEVILTFSLLFT
*Sl*TIP5;1 96 SIPMSIFYWISQMIGSVTACLLLKFTN--QQVPTHGIPQEMTGFGAAVLEGVMTFGLVYT

 H5 LE1 LE2
*Sl*TIP1;1 158 VYATAVDPKKGDLGVIAPIAIGFIVGANILAGGAFTGASM**NPA**VSFGPSLVSWTWTHQWV
*Sl*TIP1;2 159 VYATAVDPKKGDLGIIAPIAIGFIVGANILAGGAFDGASM**NPA**VSFGPAVVSWTWDNHWV
*Sl*TIP1;3 159 VYATAVDPKKGSLGTIAPIAIGFIVGANILVGGAFDGASM**NPA**VSFGPALVSWAWSNHWV
*Sl*TIP2;1 157 VYATAADPKKGSLGTIAPIAIGFIVGANILAAGPFSGGSM**NPA**RSFGPAVVSGNFAGIWI
*Sl*TIP2;2 158 VFATAADPKKGSLGTIAPIAIGLIVGANILAAGPFSGGSM**NPA**RSFGPAMVSGNFEGFWI
*Sl*TIP2;3 157 VYATAADPKKGSLGTIAPIAIGFIVGANILAAGPFSGGSM**NPA**RSFGPAVVAGDFSQNWI
*Sl*TIP2;5 181 VYATAADPKKGSLGTIAPIAIGFIVGANILAAGPFSGGSM**NPA**RSFGPAVVSGNFAGNWI
*Sl*TIP3;1 159 VYATAIDPKRGSLGIIAPLAIAFIVGANVLVGGPFEGASM**NPA**RAFGPALVGWRWRNHWI
*Sl*TIP3;2 161 VYATAVDPRRGSLSTIAPLAIAFILGANTLVGGPFEGASM**NPA**RAFGPALVGWRWRNHWI
*Sl*TIP4;1 154 VYTTLVDPKKGILEGMGPLLTGLVVGANIMAGGPFSGASM**NPA**RSFGPAFVSGIWTDHWV
*Sl*TIP5;1 154 VYA-AADPRRCVHAAIGPLAIGLMLGANVMASGPFTGGSM**NPA**YSFGSAVVKGSFGNQAV


*Sl*TIP1;1 218 YWAGPLIGGGLAGFIYEFIFISH-THEQIPS-GDF--------
*Sl*TIP1;2 219 YWLGPFGGAAIAALVYEIIFIGQNTHEQLPTTDDY--------
*Sl*TIP1;3 219 YWVGPLIGGGLAGLIYEFFFINQ-THEPLPQ------------
*Sl*TIP2;1 217 YWVGPLVGGGLAGFIYSNVFMNP-EHAPLSSDF----------
*Sl*TIP2;2 218 YWIGPLVGGSLAGLIYTNVFMTQ-EHAPLSNEF----------
*Sl*TIP2;3 217 YWVGPLIGGGLAGFIYGDVFIGCHTPLPTSEDYA---------
*Sl*TIP2;5 241 YWVGPLIGGGLAGLIYSNVFMNYGDHVPLSSDF----------
*Sl*TIP3;1 219 YWVGPFIGAAIAGIIYEFGLIQA--HDEAPVHTHHQPLAPEDY
*Sl*TIP3;2 221 YWLGPFIGAALAGLIYEYGIIQ---HETVPRPTTHQPLAPEDY
*Sl*TIP4;1 214 YWIGPFIGGGLAGFICENFFIVR-SHVPLPNEETF--------
*Sl*TIP5;1 213 YWIGPFIGAAIAGLVYDNVVFPLQVTESLRGIGGGIVAV----

Figure S2: Alignment of AA sequences of *Sl*TIP subfamily members.

Shown is an AA sequence alignment of all *Sl*TIPs. Black lines above the alignment indicate predicted transmembrane domains. The two conserved NPA motifs are shown in bold letters Residues comprising the ar/R filter are marked in grey and labelled H2, H5, LE1 and LE2. Residues occupying conserved positions one to five (from N- to C-terminus P1 to P5) are marked in yellow.
