# Supplementary material for: Genome-Wide Identification and Expression Analysis of Aquaporins in Tomato
Source: PLoS One. 2013 Nov 19;8(11):e79052. doi: 10.1371/journal.pone.0079052 (PMC3834038; doi:10.1371/journal.pone.0079052)
Supplement: Figure S3 — Alignment of AA sequences of Sl NIP subfamily members. Shown is an AA sequence alignment of all SlNIPs. Black lines above the alignment indicate predicted transmembrane domains. The two conserved NPA motifs are shown in bold letters. Residues comprising the ar/R filter are marked in grey and labelled H2, H5, LE1 and LE2. Residues occupying conserved positions one to five (from N- to C-terminus P1 to P5) are marked in yellow. A conserved Calcium-dependent protein kinase recognition site in the C-terminus is marked with blue boxes. (DOCX) [file pone.0079052.s003.docx]

*Sl*NIP1;1 1 -MADHQINVNGNINHGVSLN--IKEDHD-LNN--------HKESSS--TSSFLT------
*Sl*NIP1;2 1 -MGDQQIG--GGANGSISLN--IRDADDNLNNKNCANSVSHQDSSSNSTCSFVT------
*Sl*NIP2;1 1 ----------MESEGGNCSK--SINQNE------------LVLKEDPKSNFFQKYYRS--
*Sl*NIP2;2 1 ----------MSNLVFSM------------------------------------------
*Sl*NIP3;1 1 ---------MEEISEGIRAT-SLRIND-------CPSPLPSAIASSATPQKHLK------
*Sl*NIP3;2 1 ---------MASITS-IISTNSSKNG------IFADFSSIEEGK-HGTIQSP--------
*Sl*NIP4;1 1 MVSNKEDQITQNMEEGNVQTASNN-------------------KVG-FCSSPAV------
*Sl*NIP4;2 1 -MSTKD---IREIEEGNCSNYTNN----------------VSGDDSSLCTSPEV------
*Sl*NIP4;3 1 ----------METANIFGSQSHTNIG----------------------LGSNAG------
*Sl*NIP5;1 1 ---------MAELENGISAPATPGTP----TPLFPSLRVDS--MGSYDRKSMPR-CKCLP
*Sl*NIP6;1 1 ----------MDPEEGVSAPSTPATPGTPGAPLFGGLIKHERRNGGNGKKSLLKSCKCFG
*Sl*NIP7;1 1 -MIMK----LPSYENGLSVEFQVDAS--------------ASEQSTYDQETTSS------


*Sl*NIP1;1 41 ----------------------VPFIQKVIAEMIGTYFLIFAGCGSVVVNADKG--MITF
*Sl*NIP1;2 50 ----------------------VPFIQKIIAETLGTYFLIFAGCGSVAVNADKG--MVTF
*Sl*NIP2;1 35 -----------------------GIIKKVIAEIIATYLLVFVTCGAASLSWSDEH-KVSK
*Sl*NIP2;2 9 --------------------------Q-LVAELLGTYLSMFAGFAAMVINKKI-------
*Sl*NIP3;1 38 ------------------CFISVHFVQKLIAEFVGTYMLIFAGCAAIVLNINKNN-VVTL
*Sl*NIP3;2 36 -------------------FLSA--FQKIIAELVGTYIFIFVGCGSALVDRER---TLTI
*Sl*NIP4;1 35 ----------------------VVLGQKLIAEVIGTYFVIFAGCGSVVVNKLYGG-TITF
*Sl*NIP4;2 35 ----------------------VIIIQKVIAEAIGTYFLIFVGCGAVAVDKTYG--SVTF
*Sl*NIP4;3 23 ------------------------LAQKLFAEAIGAYVIIFAWCGSVAMYKLQDDESITF
*Sl*NIP5;1 45 LD--APTWGAPHTCLADFPAPDVSLTRKLGAEFVGTFILIFAATAGPIVNQKYNG-AESL
*Sl*NIP6;1 51 VEPWASEEGTLPAVTCMLPPPPISLARKVGAEFIGTLILIFAGTATAIVNQKTQG-SETL
*Sl*NIP7;1 36 ----NVEMLERRNVCNSILGIDPIFLRMVLAEALGTFLLMFCICGMMASMEIMGV-QVGL

 H2
*Sl*NIP1;1 77 PGVAITWGLVVMVMVYSVGHISGAHF**NPS**VTIAFASVKRFPWKQVPAYVAAQVLGATLAS
*Sl*NIP1;2 86 PGISIVWGLVVMVMVYSVGHISGAHF**NPA**VTIAFASNKRFPWKQVPAYVAAQVIGSTLAS
*Sl*NIP2;1 71 LGASVAGGLIVTVMIYAVGHISGAHM**NPA**VTFAFAAVRHFPWTQVPVYAAAQVTGAISAA
*Sl*NIP2;2 35 ----------MMVMIYTVGPVFGAHF**NPA**VTVAFASCKRVAWRNVPAYMLAQVVGATLAT
*Sl*NIP3;1 79 PGIASVWGLVVMVLIYSVGHVSGAHF**NPA**VTIAFATSKMFPWIQVPAYILVQVVGSTLAS
*Sl*NIP3;2 72 VGIALAWGLSLMALIYTLSHVSGAHF**NPA**VTIAFAAARKLPLMQVPMYVLPQFLGSTLAS
*Sl*NIP4;1 72 PGISVTWGLIVMVMVYTVGHISGAHF**NPA**VTITFSVFGRFPWKEVPFYIVAQLMGSILAS
*Sl*NIP4;2 71 PGICVAWGLIVMVMVYSVGHISGAHF**NPA**VTIAFALFRHFPVKQVPLYIMAQMVGAILGS
*Sl*NIP4;3 59 GGINMTWGAVVMVMVYSMAQVSGAHF**NPA**VTLIFTVFRRSPWKLAPVYIIAQLIGSILAG
*Sl*NIP5;1 102 IGNAACSGLAVMIVILSTGHISGAHL**NPS**LTIAFAALRHFPWVQVPAYVAAQVSASICAS
*Sl*NIP6;1 110 IGLAASTGLAVMIVILSTGHISGAHL**NPA**VTIGFAALNHFPWKHVPVYIGAQIIASFCAA
*Sl*NIP7;1 91 MEYATTAALTVVVVVFSIGPISGAHI**NPA**VTLAFAAVGHFPWSKVPLYVVAQVGGSILAT


*Sl*NIP1;1 137 GTLRLIFNGKHDHFAGTLPSG---TDFQSFVIEFIITFYLMFVISGVATDNR------AI
*Sl*NIP1;2 146 GTLRLIFNGKHDHFVGTSPTG---SDVQSLVLEFIITFYLMFVISGVATDNR------AI
*Sl*NIP2;1 131 FTLRVLLHPVTKNVGTTTPSG---SDIQALIMEIVVTFSMMFITSAVATDTK------AI
*Sl*NIP2;2 85 VTVRLMFKEEQLQFLRTILAG---TAMDKQAVSDHCLFRCKDPRTEFDLFYPSLCLLMQV
*Sl*NIP3;1 139 GSLRLIFNGKEDQFVGTVPAG---TDLQALILEFIATFYLMFVIAGVATDDR------AM
*Sl*NIP3;2 132 LTLRVLFNHQGDILPMLTQYKSPVTDFEAIFWEFLMTLILMFVICGAATDDR------AT
*Sl*NIP4;1 132 GTLSLMFDVTPEAYFGTVPVG---SDVQSLAAEIVISFLLMFVISGVGTDER------AI
*Sl*NIP4;2 131 GTLYLLLDLKTQAFFGTTPVG---TNLQSLILEFIISYLLMFVISGVATDNR------SI
*Sl*NIP4;3 119 VTLALLLD-----------------------VNPIVYFKQFF------------------
*Sl*NIP5;1 162 FALKGVFHPFMS-GGVTVPSV---NTGQAFALEFLITFNLLFVVTAVATDTR------AV
*Sl*NIP6;1 170 FTLKVVLHPIMG-GGVTVPSG---SYVQAFALEFIISFNLMFVVTAVATDTR------SV
*Sl*NIP7;1 151 YTGKLVYG-LKAEFVTTKPLH---SCTSAFFVELLATFIVLFLSASLTNYDP-----QST

H5 LE1 LE2
SlNIP1;1 188 GELAGLAVGATILLNVMFTGPISGASM**NPA**RSLGPAIVSSHYKGLWIYLVSPTLGAIAGA
SlNIP1;2 197 GELAGLAVGATVLLNVMFAGPISGASM**NPA**RSLGPAIVSSHYKGLWVYMLGPIGGAIAGA
SlNIP2;1 182 GELAGIAVGSAVCITSILAGPVSGGSM**NPA**RSIGPAMASNDYRAIWVYIIGPVCGTLLGA
SlNIP2;2 142 GELNGHVIGAVITINSILAGPISGGSI**NPT**RSLGPAILSNCYKKQWIYILGPTAGATTGI
SlNIP3;1 190 KHLSGVAIGATVSLDILFSGPLTGASM**NPA**RSLGPAIVTGHYKGLWIYIIGPTLGAIFGA
SlNIP3;2 186 KGVAGVAIGVTLVFEVLIAGPITGASM**NPA**RSLGPAIVSGVYKNQWVFVIAPILGAMTAT
SlNIP4;1 183 GHIAGIAVGMTITLNVFVVGPISGASM**NPA**RSIGPAIVRHTYKGLWVYIVGPIVGTLAGA
SlNIP4;2 182 GELAGIAIGMTILLNVLIAGPVSGASM**NPA**RSIGPAIVMHHYKGLWVYIIGPILGTICGA
SlNIP4;3 ------------------------------------------------------------
SlNIP5;1 212 GELAGIAVGATVMLNILVAGPSSGASM**NPV**RTLGPAVAAGNYKSLWIYLVAPTLGALAGA
SlNIP6;1 220 GELAGIAVGATVMLNILIAVETTGASM**NPV**RTLGPAVAVGNYKAIWIYLTAPILGALIGA
SlNIP7;1 202 GPLSGFLVGVAIGLAVLISGPVSGGSM**NPA**RSLGPAIVAWKFNNLWIYVIAPIIGAVAGV


SlNIP1;1 248 WVYNIIRFTDKPLREITK--SGSFLKSK----NSST------------------------
SlNIP1;2 257 WVYNIIRFTDKPLREITK--SGSFLKSKTLLRNPSH------------------------
SlNIP2;1 242 WSYNFIKVNDKPVQAIVPGQSFSFKLRRMKSNNHDEEQCVTL------------------
SlNIP2;2 202 WFYNAMKSVKSYNEVTKFLPFLRRLAQNKV------------------------------
SlNIP3;1 250 WTYNLMRLTNKSWGEAAKEISHSQTAIEVSSKDKVICNCGEGWSCVVSKTEAAEVGNIFF
SlNIP3;2 246 GIYGLLRQPKQ------------NTKI---------------------------------
SlNIP4;1 243 FMYNLIRATDKPLNE--LTKSVSSLRS---------------------------------
SlNIP4;2 242 FTYNLIRFTEKPLRELTLTKTSTFLKSMSRK-----------------------------
SlNIP4;3 ------------------------------------------------------------
SlNIP5;1 272 AVYTLVKLRG----DTTETPRQVRSFRR--------------------------------
SlNIP6;1 280 GVYSAVKLPDE-DRDNHPKPSLEHSFRR--------------------------------
SlNIP7;1 262 VFYRFLR-LQGWSCKPNSTPTTHQHI----------------------------------


SlNIP1;1 ------------------------------------
SlNIP1;2 ------------------------------------
SlNIP2;1 ------------------------------------
SlNIP2;2 ------------------------------------
SlNIP3;1 310 ECAEGCICIVDETSTLKKHVYVYEKTKRRKSYKMYI
SlNIP3;2 ------------------------------------
SlNIP4;1 ------------------------------------
SlNIP4;2 ------------------------------------
SlNIP4;3 ------------------------------------
SlNIP5;1 ------------------------------------
SlNIP6;1 ------------------------------------
SlNIP7;1 ------------------------------------


Supplemental Figure 3: Alignment of AA sequences of SlNIP subfamily members.

Shown is an AA sequence alignment of all *Sl*NIPs. Black lines above the alignment indicate predicted transmembrane domains. The two conserved NPA motifs are shown in bold letters. Residues comprising the ar/R filter are marked in grey and labelled H2, H5, LE1 and LE2. Residues occupying conserved positions one to five (from N- to C-terminus P1 to P5) are marked in yellow. A conserved Calcium-dependent protein kinase recognition site in the C-terminus is marked with blue boxes.
