# Supplementary material for: Genome-Wide Identification and Expression Analysis of Aquaporins in Tomato
Source: PLoS One. 2013 Nov 19;8(11):e79052. doi: 10.1371/journal.pone.0079052 (PMC3834038; doi:10.1371/journal.pone.0079052)
Supplement: Figure S4 — Alignment of AA sequences of Sl SIP subfamily members. Shown is an AA sequence alignment of all SlSIPs. The two conserved NPA motifs are shown in bold letters. Residues comprising the ar/R filter are marked in grey and labelled H2, H5, LE1 and LE2. Residues occupying conserved positions one to five (from N- to C-terminus P1 to P5) are marked in yellow. (DOCX) [file pone.0079052.s004.docx]

SlSIP1;1 1 M--GVIKAAIADGLLTFLWVFCSSNIGVSTYFIASYFGIVNEIPSLFITTLIVFVIFLMF
SlSIP1;2 1 M--GAVKAAVGDFVLTLMWVFCSSTLGIFTYLIATAFGIAQGMASLFITTVLLFMLFFVF
SlSIP1;3 1 MKMGVVKAVVADFVMTFIAIFCVSTIGVLTYIIRSAFGIAPGLASLSITILIVFLLFLML
SlSIP2;1 1 MGVSRRSLVISDFIMSFMWVWSSVLIKMFVHKILG-YG-AHDLKGEILKHAISVINMFLF

 H2
SlSIP1;1 59 DFLGDVLGGAGF**NPT**GNAAFYAAGLGDDSLVSAAVRCPAQVAGAVAGSLALVELIPKHYH
SlSIP1;2 59 GIIGDALGGAAF**NPA**GTAAFYAAGVGKDSLFTVATRFPAQAAGAVAGAVAILEVIPTQYK
SlSIP1;3 61 SVIAEALGGAAF**NPA**ATAAFYAAGVGKDSLFSVAARFPAQ--------------------
SlSIP2;1 59 ALLTKATKGGAY**NPL**TILSGAISGDLTNFIFTVAARIPSQVFGSITG----VRFIIAAFP

 H5
SlSIP1;1 119 HMLDGPALKVDVQTGAIAEGVLTFVITFMIFVIVLRGPESVLLKNWLLTMVTLPLVLAGS
SlSIP1;2 119 HMLGGPSLKVDLHNGAIAEGILTFVMTFLVFIIVLKGPKSALLKNWLLAMSTVTMVVAGS
SlSIP1;3 101 ---------------------------YLLL-----------------------------
SlSIP2;1 115 NIGRGPVLSIDIHRGALTEGILTFAIVSISLGLSRRSRASTFMKTWISSLSKLTLHILGS

 LE1 LE2
SlSIP1;1 179 NFTGPSM**NPA**NAFGWAYLSNTHKTLEHFYVYWISPFIGAILAAWIFRVLFPPPVEQK-PQ
SlSIP1;2 179 KYTGPSM**NPA**NAFGWAYINNMHNTWEQFYVYWICPFVGAIMAAWTFRAVFPAPAKKKKPQ
SlSIP1;3 ------------------------------------------------------------
SlSIP2;1 175 DLTGGCM**NPA**SVMGWAYARGDHITKEHIHVYWLAPIQATLLAVWTFNLLVSPSKDKEAKK


SlSIP1;1 238 KQKRN-
SlSIP1;2 239 KKKRN-
SlSIP1;3 ------
SlSIP2;1 235 TEKKSE


Figure S4: Alignment of AA sequences of SlSIP subfamily members.

Shown is an AA sequence alignment of all *Sl*SIPs. The two conserved NPA motifs are shown in bold letters. Residues comprising the ar/R filter are marked in grey and labelled H2, H5, LE1 and LE2. Residues occupying conserved positions one to five (from N- to C-terminus P1 to P5) are marked in yellow.
