# Supplementary material for: Genome-Wide Identification and Expression Analysis of Aquaporins in Tomato
Source: PLoS One. 2013 Nov 19;8(11):e79052. doi: 10.1371/journal.pone.0079052 (PMC3834038; doi:10.1371/journal.pone.0079052)
Supplement: Figure S5 — Alignment of AA sequences of Sl XIP subfamily members. Shown is an AA sequence alignment of all SlXIPs. The two conserved NPA motifs are shown in bold letters. Residues comprising the ar/R filter are marked in grey and labelled H2, H5, LE1 and LE2. Residues occupying conserved positions one to five (from N- to C-terminus P1 to P5) are marked in yellow. Note that for SlXIP1;2′ the deduced AA sequence from a corrected EST is shown (see main text). (DOCX) [file pone.0079052.s005.docx]

*Sl*XIP1;1 1 ------------------------------------------------------------
*Sl*XIP1;2 1 ------------------------------------------------------------
*Sl*XIP1;3 1 ------------------------------------------------------------
*Sl*XIP1;4 1 ------------------------------------------------------------
*Sl*XIP1;5 1 ------------------------------------------------------------
*Sl*XIP1;6 1 MAIRMPRIIKKSSDIPKGHFVVYVGEKQKKRFVIPISFLSEPLFQDLLNQAEEEFGFDHP


*Sl*XIP1;1 1 ------------------------------------------------------------
*Sl*XIP1;2’ 1 ------------------------------------------------------------
*Sl*XIP1;3 1 ------------------------------------------------------------
*Sl*XIP1;4 1 ------------------------------------------------------------
*Sl*XIP1;5 1 ------------------------------------------------------------
*Sl*XIP1;6 61 MGGVTIPCTEDLFVNLTSRLRNLHLGYVPVLVDSVADTNRHKAHMVLMMRGPLFQMAIRV


*Sl*XIP1;1 1 ------------------------------------------------------------
*Sl*XIP1;2’ 1 ------------------------------------------------------------
*Sl*XIP1;3 1 ------------------------------------------------------------
*Sl*XIP1;4 1 ------------------------------------------------------------
*Sl*XIP1;5 1 ------------------------------------------------------------
*Sl*XIP1;6 121 PRIIKKSSTSLQVPKGHFAVYVGEKQKKRFVIPISYLSQSSFQDLLSQAEEEFGFDHPMG


*Sl*XIP1;1 1 ------------MASNSNVVFGDEESQISGG-TNRVQPCSSTPRKINTIDDDEGKKHNFL
*Sl*XIP1;2’ 1 ------------MASKNNVVFGDEENQLPGG-TNKVQPCSSTSRK-NTIDD-EGKKPIFL
*Sl*XIP1;3 1 ------------MFSN------------------------TQRFKLNNIDD-DGKKPNFL
*Sl*XIP1;4 1 ------------MGSNNNAVFGDEENQFSCG-TNKVQPCSSTPKR-STIDD-EGKKLNFL
*Sl*XIP1;5 1 ------------MGSNNNIFLGDEESQLSAGNTNRVQPYSSTPKK-NTIDD-EGKKQNFL
*Sl*XIP1;6 181 GVTIPCPEDIFIMDSTNGNVLGDEESQLSFGSSNKIQPTISITQKQVPSSDDEKKKYTCL

 H2
*Sl*XIP1;1 48 SLSQRLGVADFFCLDVWRASMGELLGSAVLVFMLDTIVISTFESETKMPNLIMSILIAVV
*Sl*XIP1;2’ 46 SFSERLGVPDFFCLDVWRASMGELLGSAVLVFMLDTIVISTLESDTKMPNLIMSILIAIV
*Sl*XIP1;3 24 SI---LGVPGFFCLDVWRASMGELIGSAVLVFMLDTIVISTLESDTKMPNLIMSILIAIV
*Sl*XIP1;4 46 SFSERLGVSDFFSLDVWRASIGELLGTAVLVFMLDTIVISTLESDIKMPNLILSILAAVI
*Sl*XIP1;5 47 SLSQRLGVPDFFSLDVWRASMGELLGSAVLVFMVDTIVISTSESDAEMPNLIMSILLAIV
*Sl*XIP1;6 241 TMAERLGLPDFFSLDVWRASVGELLGSAVLVFMLDTVVISTLESDVKMPNLIMSILIAIT


*Sl*XIP1;1 108 ITILLLAVVPVSGGHI**NPV**ISFSAALVGIISMSRAIIYIVAQCVGAILGALALRAVVSSS
*Sl*XIP1;2’ 106 ITILLLAVVPVSGGHI**NPI**ISFSAALVGIISMSRAIIYIMAQCVGAILGALALRAVVSSS
*Sl*XIP1;3 81 ITILLLAVVPVSGGHI**NPI**ISFSAALVGIISMSRAIIYIMAQCVGAILGALALRAVVSSS
*Sl*XIP1;4 106 ITILLLAVVPVSGGHI**NPV**ISFSAALVGIISMSRAIIYIMAQCLGAILGALALKAVVSSS
*Sl*XIP1;5 107 LIVLLLAVVPVSGGHL**NPV**ISFSAALVGIISMSRAIIYIMAQCLGAVLGALALKAVVSST
*Sl*XIP1;6 301 LTILILAVFPVSGGHI**SPV**ITFSSALVGLISMSRAIIYIVAQCVGAILGALALKAVLSST


*Sl*XIP1;1 168 IEDTFSLGGCTVTIIAPGPNGPVIVGLETAQALWLEIFCTFVFLFASIWMAYDHRQAKAL
*Sl*XIP1;2’ 166 IEDTFSLGGCTITIIAPGPNGPVIVGLEMAQALWLEIFCTFVFLFASIWMAYDHRQAKSL
*Sl*XIP1;3 141 IEDTFSLGGCTITIIASGPNGPVIVGLEMAQALWLEIFCTFVFLFASIWMAYDHRQAKAL
*Sl*XIP1;4 166 IEDTFSLGGCTITIIAPGPNGPISVGLETAQALWLEIFCTFVFLFASIWMAYDHRQAKAL
*Sl*XIP1;5 167 IEDTFSLGGCTITIIAPGPNGPVTLGLETAQALWLEIFCTFVLLFASIWMAYDHRQAKAL
*Sl*XIP1;6 361 IEQRFSLGGCTVTVVTPGLNGPEIVGLEIAQAFWLEFMCTFALLFGSLWMAYDHRQSKAL

 H5 LE1 LE2
*Sl*XIP1;1 228 GHVTVLSIVGLVLGLLVFISTTVTAKKGYGGAGI**NPA**RCLGPAIIRGGHLWDGHWIFWVG
*Sl*XIP1;2’ 226 GHVTVLSIVGVVLGLLVFISTTNTARKGYGGAGM**NPA**RCLGPAIIRGGHLWDGHWIFWVG
*Sl*XIP1;3 201 GLVTVLSIVGLVLGLLVFISTTITAKRGYGGAGM**NPA**RCLGPALVRGGHLWDGHWIFWVG
*Sl*XIP1;4 226 GHVTVLSIVGVVLGLLVFISTTVTAKKGYAGAGM**NPA**RCFGPAIVRGGHLWDGHWIFWVG
*Sl*XIP1;5 227 GLVTVLTIVGLVLGLLVFISTTVTTKRGYAGVGM**NPA**RCLGPAIVRGGHLWDGHWIFWIG
*Sl*XIP1;6 421 GLITVMSIVGLLAGILVFISTSVTAKKGYAGAGM**NPA**RCFGAAFVRGGHLWNGHWIFWVG

*Sl*XIP1;1 288 PTIGCVAFYVYTKIIPTKHFLAEYGFKHDFVGVVKAL--SNV
*Sl*XIP1;2’ 286 PTIGCVAFYVYTKIIPPKHFLGEYGFKHDFVGVVKAL--SNV
*Sl*XIP1;3 261 PTIGCVAFYVYTKIIPAEHFNAEYGYKHDFVGVVKALFGSNV
*Sl*XIP1;4 286 PIIGCVAFYVYTKIIPTKHFNAEYGYKHDFVGVVKALVGSNV
*Sl*XIP1;5 287 PTISCLAFYVYTKIIPPKHFHAEYGYKHDFVGVVKALFESNV
*Sl*XIP1;6 481 PGLACFAFYFYTKIIPSDHFQTD-GYKHDFLAIIETLFNQR-


Figure S5: Alignment of AA sequences of *Sl*XIP subfamily members.

Shown is an AA sequence alignment of all *Sl*XIPs. The two conserved NPA motifs are shown in bold letters. Residues comprising the ar/R filter are marked in grey and labelled H2, H5, LE1 and LE2. Residues occupying conserved positions one to five (from N- to C-terminus P1 to P5) are marked in yellow. Note that for *Sl*XIP1;2’ the deduced AA sequence from a corrected EST is shown (see main text).
