# Supplementary material for: Genome-Wide Identification and Expression Analysis of Aquaporins in Tomato
Source: PLoS One. 2013 Nov 19;8(11):e79052. doi: 10.1371/journal.pone.0079052 (PMC3834038; doi:10.1371/journal.pone.0079052)
Supplement: Figure S6 — Phylogenetic analysis of aquaporins from tomato and 13 other species. Shown is a phylogenetic tree from an alignment of AA sequences from all identified MIPs from Solanum lycopersicum together with MIPs from Arabidopsis thaliana and Oryza sativa. For the XIP subfamily sequences from Physcomitrella patens, Populus trichocarpa, Ricinus communis, Gossypium hirsutum, Gossypium raimondii, Lactuca scariola, Citrus clementine, Citrus sinensis, Ipomoea nil, Solanum tuberosum and Nicotiana tabacum were used. For tomato the gene name and the best hit EST are given. If no EST was found the locus is given. For Arabidopsis and rice the gene name and the locus are given; for other species the NCBI accession number or the JGI protein ID is given. Bold font indicates tomato MIPs. #1 indicates EST is not full length. #2 indicates EST contained a frameshift leading to premature termination; Putative full-length AA sequence was used. (DOCX) [file pone.0079052.s006.docx]

Figure S6: Phylogenetic analysis of aquaporins from tomato and 13 other species.

Shown is a phylogenetic tree from an alignment of AA sequences from all identified MIPs from *Solanum lycopersicum* together with MIPs from *Arabidopsis thaliana* and *Oryza sativa*. For the XIP subfamily sequences from *Physcomitrella patens*, *Populus trichocarpa*, *Ricinus communis*, *Gossypium hirsutum*, *Gossypium raimondii*, *Lactuca scariola*, *Citrus clementine*, *Citrus sinensis*, *Ipomoea nil*, *Solanum tuberosum* and *Nicotiana tabacum* were used. For tomato the gene name and the best hit EST are given. If no EST was found the locus is given. For Arabidopsis and rice the gene name and the locus are given; for other species the NCBI accession number or the JGI protein ID is given. Bold font indicates tomato MIPs. #^1^ indicates EST is not full length. #^2^ indicates EST contained a frameshift leading to premature termination; Putative full-length AA sequence was used.
