# Supplementary material for: Genome-Wide Identification and Expression Analysis of Aquaporins in Tomato
Source: PLoS One. 2013 Nov 19;8(11):e79052. doi: 10.1371/journal.pone.0079052 (PMC3834038; doi:10.1371/journal.pone.0079052)
Supplement: Table S1 — Sequences of oligonucleotides and PCR program settings used for gene expression analysis. Shown are the sequences of the forward (FWD) and the (REV) primer used to analyze the expression of each SlAQP. Below each primer pair the PCR program used for each target gene is given. (DOCX) [file pone.0079052.s007.docx]

Table S1: Sequences of oligonucleotides and PCR program settings used for gene expression analysis

Shown are the sequences of the forward (FWD) and the (REV) primer used to analyze the expression of each *Sl*AQP. Below each primer pair the PCR program used for each target gene is given.

|  | Orientation | Sequence |
| --- | --- | --- |
| *SlPIP1;1* | FWD | 5’- GAAATCTTAGTGAGTGAGTGAG -3’ |
|  | REV | 5’- ATGATGATAGTTCACCAGG -3’ |
|  | 95°C 20s, {95°C 15s, 62°C 20s} 30 cycles | |
| *SlPIP1;2* | FWD | 5’- CTATTGGCTATAGCTATGT -3’ |
|  | REV | 5’- CTATTGGCTATAGCTATGT -3’ |
|  | 95°C 20s, {95°C 15s, 52°C 20s, C 20s} 30 cycles | |
| *SlPIP1;3* | FWD | 5’- GGCTACCATTCCAATCACCG -3’ |
|  | REV | 5’- CAACAGCACCAGACAGG -3’ |
|  | 95°C 20s, {95°C 15s, 58°C 20s, C 20s} 25 cycles | |
| *SlPIP1;5* | FWD | 5’- GCTGCTCTTGCTGCTATTT -3’ |
|  | FWD | 5’- CCTTCATTGATAAGGTACA -3’ |
|  | 95°C 20s, {95°C 15s, 59°C 20s, 72°C 20s} 45 cycles | |
| *SlPIP1;7* | FWD | 5’- CACTCACTAACTCCCATC -3’ |
|  | REV | 5’- CGTTGAACATTTGGAATCGG -3’ |
|  | 95°C 20s, {95°C 15s, 62°C 20s} 40 cycles | |
| *SlPIP2;1* | FWD | 5’- GTGCTGCTGTTGTTTATGGACA -3’ |
|  | REV | 5’- CATCCAACACAACTCTAACAAC -3’ |
|  | 95°C 20s, {95°C 15s, 60°C 20s} 30 cycles | |
| *SlPIP2;4* | FWD | 5’- CAATGGTGACAAGGCGTGG -3’ |
|  | REV | 5’- GAAGGCGAATTCATAGGAT -3’ |
|  | 95°C 20s, {95°C 15s, 61°C 20s} 30 cycles | |
| *SlPIP2;5* | FWD | 5’- GGATATGGAGTATGGAAATG -3’ |
|  | REV | 5’- TTGGTCACCATCACTTTG -3’ |
|  | 95°C 20s, {95°C 15s, 62°C 20s} 35cycles | |
| *SlPIP2;6* | FWD | 5’- CAGAGCATCCTCTGTTT -3’ |
|  | REV | 5’- CACCGCAAATATCGCCTC -3’ |
|  | 95°C 20s, {95°C 15s, 57°C 20s} 30 cycles | |
| *SlPIP2;8* | FWD | 5’- GGAGCTGCTGTTATTGCTGA -3’ |
|  | REV | 5’- GCACAGATCCAAGGCTAAGA -3’ |
|  | 95°C 20s, {95°C 15s, 62°C 20s} 35 cycles | |
| *SlPIP2;9* | FWD | 5’- GCAATGGCAGCAGCAATATACCA -3’ |
|  | REV | 5’- CGAAAGAGAATAGACCACCA -3’ |
|  | 95°C 20s, {95°C 15s, 64°C 20s} 30 cycles | |
| *SlPIP2;12* | FWD | 5’- AGTAGTGCATTGCCGGAGC -3’ |
|  | REV | 5’- CATGGATCATGATCACTTTCACTC -3’ |
|  | 95°C 20s, {95°C 15s, 66°C 20s} 35 cycles | |
| *SlTIP1;1* | FWD | 5’- GTGCCTTTACTGGAGCTTCAAT -3’ |
|  | REV | 5’- GTACAATTCAACAGTGCCCA -3’ |
|  | 95°C 20s, {95°C 15s, 63°C 20s} 25 cycles | |
| *SlTIP1;2* | FWD | 5’- GTAGGAGGTCACATTACACT -3’ |
|  | REV | 5’- CGCACCACCAGCCAAGATAT -3’ |
|  | 95°C 20s, {95°C 15s, 63°C 20s} 30 cycles | |
| *SlTIP2;1* | FWD | 5’- CACTTGTTGGCGGTGGGTTA -3’ |
|  | REV | 5’- CATGAATGTACAGCTGCAA -3’ |
|  | 95°C 20s, {95°C 15s, 62°C 20s} 35 cycles | |
| *SlTIP2;2* | FWD | 5’- GTCCATTAGTTGGTGGTAGTT -3’ |
|  | REV | 5’- GGATGTACAGCTGCAAACCA -3’ |
|  | 95°C 20s, {95°C 15s, 62°C 20s} 35 cycles | |
| *SlTIP2;3* | FWD | 5’- GCTCACTTGGAACCATTGC -3’ |
|  | REV | 5’- CACTGTTGAAGACTTGTTC -3’ |
|  | 95°C 20s, {95°C 15s, 63°C 20s} 45 cycles | |
| *SlTIP3;1* | FWD | 5’- GATAAGTTGTACCCTGATAGAGC -3’ |
|  | REV | 5’- CATTGCCAACTCCTGATGCTA -3’ |
|  | 95°C 20s, {95°C 15s, 64°C 20s, C 20s} 30 cycles | |
| *SlTIP3;2* | FWD | 5’- GTTGGAGGTGGAGGAACCA -3’ |
|  | REV | 5’- GATGTGCAGGCAGCTACATACA -3’ |
|  | 95°C 20s, {95°C 15s, 68°C 20s} 45 cycles | |
| *SlTIP4;1* | FWD | 5’- CCACTTCTAACTGGGCTTGTTG -3’ |
|  | REV | 5’- CATGCATAGATAAGGTAGACTTCC -3’ |
|  | 95°C 20s, {95°C 15s, 64°C 20s} 45 cycles | |
| *SlNIP1;2* | FWD | 5’- AAGGCGCTGAACGGACAC -3’ |
|  | REV | 5’- CAAATCCAAAACCCTACTACG -3’ |
|  | 95°C 20s, {95°C 15s, 64°C 20s} 45 cycles | |
| *SlNIP2;1* | FWD | 5’- GGAGCATGGTCCTATAATTTCAT -3’ |
|  | REV | 5’- CTTATTCACCATCTATCCGACAC -3’ |
|  | 95°C 20s, {95°C 15s, 64°C 20s, C 20s} 45 cycles | |
| *SlNIP3;1* | FWD | 5’- TCTGAAGGAATTCGAGCAAC -3’ |
|  | REV | 5’- TTTGCACGAAATGTACGC -3’ |
|  | 95°C 20s, {95°C 15s, 62°C 20s} 40 cycles | |
| *SlNIP4;1* | FWD | 5’- TGGAGGAGGAAGTCTTTGATAC -3’ |
|  | REV | 5’- CCTTCTTCCATATTCTGAGTG -3’ |
|  | 95°C 20s, {95°C 15s, 60°C 20s} 40 cycles | |
| *SlNIP5;1* | FWD | 5’- CATCAAGGTTACTCAATTCTGC -3’ |
|  | REV | 5’- GATAAATCAGAATACAACAACTG -3’ |
|  | 95°C 20s, {95°C 15s, 60°C 20s} 45 cycles | |
| *SlNIP6;1* | FWD | 5’- CACCTGGAAGGCAAGAGTGGG -3’ |
|  | REV | 5’- CCACTCATGTGGCACTAGTTCTG -3’ |
|  | 95°C 20s, {95°C 15s, 68°C 20s, 72°C 20s} 45 cycles | |
| *SlSIP1;1* | FWD | 5’- GTACTACTGATTCAAGTAGG-3’ |
|  | REV | 5’- GGCAGCATTTCCAGTTGGATTGA-3’ |
|  | 95°C 20s, {95°C 15s, 59°C 20s, C 20s} 45 cycles | |
| *SlSIP1;2* | FWD | 5’- GATAGACTCTTCTGCTTCAG-3’ |
|  | REV | 5’- CACAAGCACAACTCAAAACAG-3’ |
|  | 95°C 20s, {95°C 15s, 62°C 20s, C 20s} 30 cycles | |
| *SlSIP2;1* | FWD | 5’- CTGGTTCAGAAACTACATG -3’ |
|  | REV | 5’- CTTGAAGTTACTGGCATCC -3’ |
|  | 95°C 20s, {95°C 15s, 55°C 20s, 72°C 20s} 30 cycles | |
| *SlXIP1;1* | FWD | 5’- TCATGCACACAATTTCAGG -3’ |
|  | REV | 5’- AAATCTGGCTTTCCTCATC -3’ |
|  | 95°C 20s, {95°C 15s, 60°C 20s} 40 cycles | |
| *SlXIP1;2* | FWD | 5’- GCCTATGGTGATGTATGG -3’ |
|  | REV | 5’- AACTTGTACAACCGAAAGAC -3’ |
|  | 95°C 20s, {95°C 15s, 62°C 20s} 35 cycles | |
| *SlXIP1;3* | FWD | 5’- GATCCCATCGAGTATCAAAATC -3’ |
|  | REV | 5’- AGGAACGCCCAGTATTGAAAG -3’ |
|  | 95°C 20s, {95°C 15s, 55°C 20s, 72°C 20s} 38 cycles | |
| *Ubiquitin* | FWD | 5’- CACCAAGCCAAAGAAGATCA -3’ |
|  | REV | 5’- TCAGCATTAGGGCACTCCTT -3’ |
|  | 95°C 20s, {95°C 15s, 55°C 20s, 72°C 20s} 35 cycles | |
